# Demethylation and derepression of genomic retroelements in the skeletal muscles of aged mice

**DOI:** 10.1111/acel.13042

**Published:** 2019-09-27

**Authors:** Byungkuk Min, Kyuheum Jeon, Jung Sun Park, Yong‐Kook Kang

**Affiliations:** ^1^ Development and Differentiation Research Center Korea Research Institute of Bioscience Biotechnology (KRIBB) Daejeon Korea; ^2^ Department of Functional Genomics University of Science and Technology (UST) Daejeon Korea

**Keywords:** aging, DNA methylation, endogenous retrovirus (ERV), LINE1, MBD‐seq, retroelement, skeletal muscle

## Abstract

Changes in DNA methylation influence the aging process and contribute to aging phenotypes, but few studies have been conducted on DNA methylation changes in conjunction with skeletal muscle aging. We explored the DNA methylation changes in a variety of retroelement families throughout aging (at 2, 20, and 28 months of age) in murine skeletal muscles by methyl‐binding domain sequencing (MBD‐seq). The two following contrasting patterns were observed among the members of each repeat family in superaged mice: (a) hypermethylation in weakly methylated retroelement copies and (b) hypomethylation in copies with relatively stronger methylation levels, representing a pattern of “regression toward the mean” within a single retroelement family. Interestingly, these patterns depended on the sizes of the copies. While the majority of the elements showed a slight increase in methylation, the larger copies (>5 kb) displayed evident demethylation. All these changes were not observed in T cells. RNA sequencing revealed a global derepression of retroelements during the late phase of aging (between 20 and 28 months of age), which temporally coincided with retroelement demethylation. Following this methylation drift trend of “regression toward the mean,” aging tended to progressively lose the preexisting methylation differences and local patterns in the genomic regions that had been elaborately established during the early period of development.

## 1 INTRODUCTION

Both muscle mass and strength start to decline around middle age, and the rate of decline accelerates with age (Hughes et al., 2001). Although the mechanisms underlying skeletal muscle aging are not yet fully understood, it is generally believed that changes in DNA methylation and in other epigenetic modifications have a huge influence on the aging process and likely contribute to aging phenotypes. The maintenance of certain DNA methylation patterns across the genome is necessary to sustain gene expression programs that are specific for terminally differentiated or differentiating cells, including skeletal muscle cells. In reality, DNA methylation is implicated in muscle development and differentiation (Carrio & Suelves, 2015), and several methylome studies have shown chronological alterations in DNA methylation during myogenic differentiation from cultured myoblasts to a myotube (Tsumagari et al., 2013). Furthermore, many genes with muscle‐specific expression, including *MYOD* (Brunk, Goldhamer, & Emerson, 1996), *MYOG* (Fuso et al., 2010), *α‐SMA* (Hu, Gharaee‐Kermani, Wu, & Phan, 2010), and *SIX1* (Wu et al., 2011), are transcriptionally regulated by de novo methylation and demethylation; for example, the gene expression changes after treatment with 5‐azacytidine (Montesano, Luzi, Senesi, & Terruzzi, 2013) or *DNMT1* antisense cDNA (Szyf, Rouleau, Theberge, & Bozovic, 1992).

Comprehensive DNA methylation analyses have been facilitated by genome‐wide sequencing technology that enables the detection of numerous methylation alterations that are present in aged tissues. However, methylome research on skeletal muscle aging in mammals has been scarce, and no consensus on the relationship between them has been reached. Zykovich et al. (2014) compared the DNA methylation patterns of human skeletal muscles from healthy young (~20 years of age) and aged (~80 years of age) males and observed predominant hypermethylation patterns in the aged skeletal muscles. Day et al. (2013) explored DNA methylation changes with age in various tissues and found that skeletal muscle tissues exhibited the strongest association with tissue‐specific genes and the least overlap in the list of age‐associated CpG dinucleotides (CpGs) with other tissues. Jin et al. (2014) compared the skeletal muscle methylation of young and middle‐aged pigs and found that there was a global hypomethylation trend in middle‐aged pigs. In contrast to the former two human studies that utilized bead chip arrays, which are used to perform limited examinations of the selected CpGs around the genic regions, the latter pig study utilized a genome‐wide analysis technique called methylated DNA immunoprecipitation (MeDIP), but their analyses were also restricted only to the genic regions. Consequently, all of these skeletal muscle methylome studies on age‐associated methylation drift lacked information about the nongenic regions, including retroelements.

Aging is associated with the loss of repressive heterochromatin integrity, leading to the abnormal activation of gene expression in those regions (Park et al., 2017; Villeponteau, 1997). This heterochromatin loss with age, a hallmark of aging occurring in species ranging from yeast to humans (Haithcock et al., 2005; Larson et al., 2012; Tsurumi & Li, 2012; Villeponteau, 1997), makes the genome unstable, and this genome instability is furthered by the increased expression and transposition of a variety of retroelements (Pal & Tyler, 2016). Sedivy et al. proposed an “aging‐by‐transposition” model, in which the retroelements and their transposases are the primary causes of structural dysregulation in the genome to manifest the aging phenotypes (Sedivy et al., 2013). It is notable that retroelement expression and transposition are also associated with cancer development (Kang, 2018; Lee et al., 2012) and neurodegenerative diseases (Reilly, Faulkner, Dubnau, Ponomarev, & Gage, 2013; Tan et al., 2012).

With some exceptions, aging in mammals is more commonly associated with CpG hypomethylation, particularly at repeats (Bjornsson et al., 2008; Bollati et al., 2009; Bormann et al., 2016; Horvath, 2013; Jintaridth & Mutirangura, 2010). Since the loss of DNA methylation at genomic repeats will intensify the risk of transposition, it may be, at least partly, causally related to the loss of heterochromatin during aging. However, the age‐associated methylomic changes at genomic repeats have not been extensively studied. In the present study, we analyzed the methylomes of aged skeletal muscle tissues with a keen interest in retroelement sequences; our aim was to evaluate the epigenetic stability during skeletal muscle aging. We used methyl‐CpG‐binding domain (MBD)‐based capture sequencing, which is relevant to look into whole genomic repeats and other nongenic chromosomal regions that have never been explored before in conjunction with skeletal muscle aging. We observed that most retroelement copies, particularly those large‐sized copies (>5 kb), were evidently demethylated in the skeletal muscles of superaged mice, and the clustering analysis uncovered two contrasting patterns of DNA methylation shifts within the related genomic repeats. We consider these global, age‐related methylation changes to be one of the epigenomic characteristics of skeletal muscle aging.

## 2 RESULTS

### 2.1 Statistics of MBD capture sequencing

We examined the epigenetic stability of skeletal muscle DNA during aging. The genomic DNA was extracted from the skeletal muscles of 2‐month‐old (young, 2 m), 20‐month‐old (aged, 20 m), and 28‐month‐old (superaged, 28 m; *n* = 3 each group) mice. With the genomic DNAs pooled by age, the MBD‐based capture was performed to enrich methylated DNA fragments. Using the resulting DNA fragments, we performed high‐throughput Illumina sequencing and obtained 62 million reads per age group on average, and the alignment efficiency rates ranged from 95.6% to 97.4% (Table S1). The average GC content of the reads was ~65% in all groups (data not shown). This high GC content indicates the successful enrichment of methylated DNAs during MBD capture. The T‐cell MBD‐seq data, which were included for comparison, showed similar features. T cells were chosen because they are implicated in chronic inflammation, which is known as the most important contributor to sarcopenia (Coelho Junior et al., 2016; Wilson, Jackson, Sapey, & Lord, 2017), a muscle disease (muscle failure) rooted in adverse muscle changes that accrue across a lifetime (Cruz‐Jentoft et al., 2019).

### 2.2 A perturbation in the methylomic structures of superaged skeletal muscle DNA

**Figure 1:**
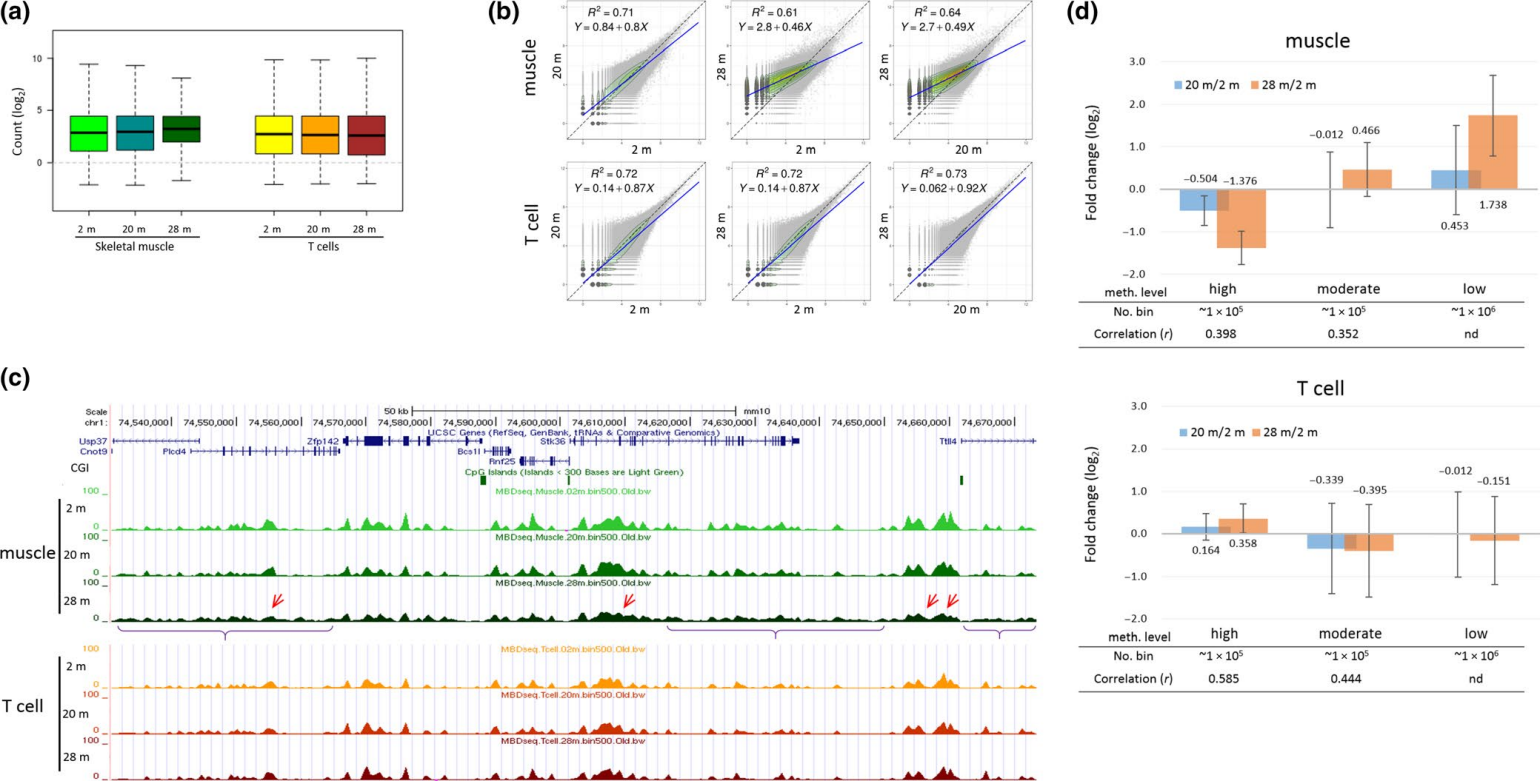
A bimodal pattern of change in global DNA methylation states during murine skeletal muscle aging. (a) Box plots showing distributions of read counts (log_2_ scale) in 500‐bp bins obtained by MBD capture sequencing of skeletal muscle and T cells in 2‐, 20‐, and 28‐month‐old mice (2, 20, and 28 m, respectively; *n* = 3 each). (b) DNA methylome correlations between age groups. Each *x*‐ and *y*‐axis indicates the read counts in log_2_ scale. Coefficient of determination (*R^2^*) and the equation for each linear regression line (blue) are denoted. (c) The browser view of DNA methylation of a randomly chosen genomic region (chr1:74,540,000–74,670,000) in mice of different ages. Arrows and brackets indicate the observed loci of demethylation (loss of methylation sites, LOM) and noisy methylation (gain of methylation sites, GOM), respectively, in the genome browser. Notably, CpG islands (CGIs) are devoid of methylation signals. (d) A bimodal pattern of methylation change during skeletal muscle aging. Fold changes on the log_2_ scale of methylation levels of 20‐m and 28‐m muscles relative to 2‐m muscle in top‐ranked high‐level methylation (*n* = 100,000), mid‐ranked moderate‐level methylation (*n* = 100,000; average read count ≅ 10), and bottom‐ranked low‐level methylation regions (*n* = 1,048,575) were calculated after sorting the genomic regions (bins) in 2‐m muscle by methylation level. Error bars: standard deviation. nd, not determined; *r*, Pearson's correlation

Box plots show the distributions of the read counts in 500‐bp bins and reveal that there are increased mean methylation levels and decreased variance among the genomic regions in 28‐m muscle (Figure 1a). Histograms for the distribution of bins by fold‐change values between the age groups showed a shift to hypermethylation in the 28‐m muscle (Figure S1). In the scatter plots comparing the methylation levels between the age groups, there was a perturbation in the methylomic structure as the slope of regression line was noticeably reduced in the matches of the 28‐m muscle versus the 2‐m or 20‐m muscle (Figure 1b). This change was not evident in T cells. Figure 1c shows a representative genome browser view of a randomly chosen genomic region. Basically, both skeletal muscles and T cells possessed similar methylation profiles in all age groups, but notwithstanding the resemblance, the local events of the loss of methylation (LOM) and the gain of methylation (GOM) appeared only in the skeletal muscles of superaged mice.

To measure the extent of the methylation shift during aging, we examined the genomic regions with methylation levels at all values: top‐ranked (high‐level methylation; ~1 × 10^5^ bins), bottom‐ranked (low‐level methylation; ~1 × 10^6^ bins), or intermediate (~10 read counts; ~1 × 10^5^ bins) after sorting the bins in 2‐m muscles by their methylation levels. The high‐methylation regions in the skeletal muscle showed an obvious LOM that was intensified with increasing age (*p* ~ 0, two‐sample *t* test; Figure 1d). Conversely, both the low and moderate methylation regions showed GOM. These alterations accelerated with age; in the high‐methylation regions, the rate of methylation reduction was approximately 0.07‐fold per month (1.38‐fold/20) up to the 20th month and was 0.093‐fold (~2.6/28) up to the 28th month (Figure 1d), indicating a steep rise of the reduction rate with age. A similar acceleration of the rate of methylation increase was observed in the low‐methylation regions (0.068‐ and 0.119‐fold, respectively). The T cells did not undergo such a clear change in the aged mice.

### 2.3 Two contrasting patterns of methylation drift among the members of each repeat family

**Figure 2:**
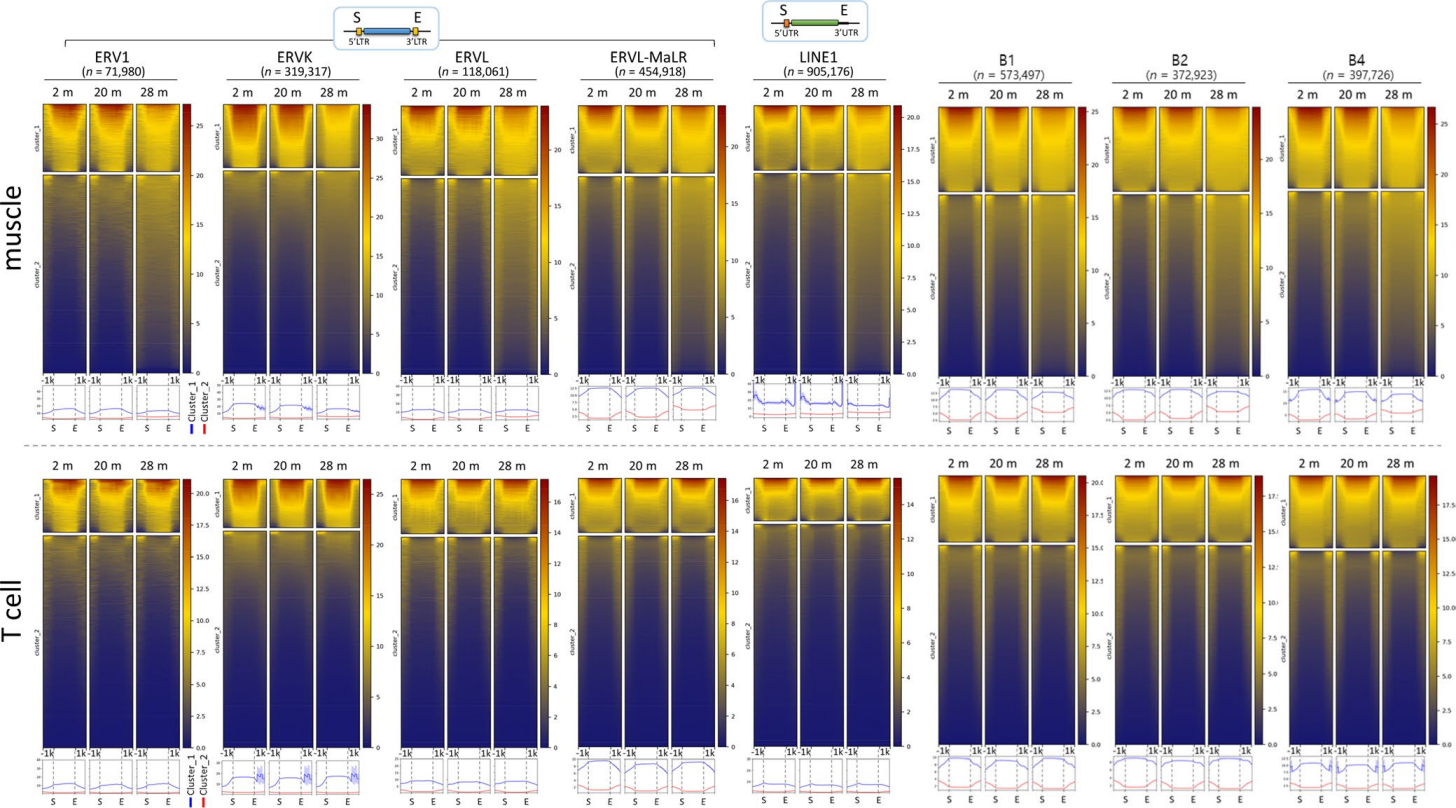
Age‐associated changes in global DNA methylation in retroelements and single‐copy sequences. Heatmaps of DNA methylation densities are shown within the start (S) and the end (E) sites of various retroelement families. Each group was subdivided by methylation level into two unsupervised clusters—cluster 1 (higher methylation) and cluster 2 (lower methylation)—and the mean methylation levels of the two clusters (blue and red lines, respectively) were examined separately, as shown in the graphs at the bottom. The numbers (n) of the members of each group are indicated. Schematic structures of ERVs and LINE1s are presented above

Genomic repeats are known to commonly undergo demethylation during aging (Pal & Tyler, 2016), which is in contrast to our result of the overall increase in DNA methylation in the 28‐m skeletal muscle. We examined the methylation states of various genomic repeats, including endogenous retroviruses (ERVs; ERV1, ERVK, ERVL, and MaLR) and long and short interspersed nuclear elements (LINEs and SINEs). We observed an age‐dependent increase in the DNA methylation levels in all of these repeat families (Figure S2). Clustering by the *k*‐means algorithm (*k* = 2) classified the members in each repeat family into highly (cluster 1) and weakly (cluster 2) methylated copies (Figure 2). We found that the elements in cluster 2 of the 28‐m muscle were hypermethylated. More interestingly, in all repeat families in the muscle, the two clusters displayed an opposite pattern of methylation changes: an LOM in cluster 1 and a GOM in cluster 2. This contrasting change was especially prominent in the ERV1, ERVK, LINE1, and SINE B1, B2, and B4 sequences. On the other hand, the ERVL and ERVL‐MaLR sequences exhibited a GOM pattern only without a recognizable LOM, the significance of which is unknown at present. In contrast to the skeletal muscle tissues, the T cells stably maintained their methylation states in these genomic repeats regardless of age.

### 2.4 A sharp demethylation of large retroelements in skeletal muscle

**Figure 3:**
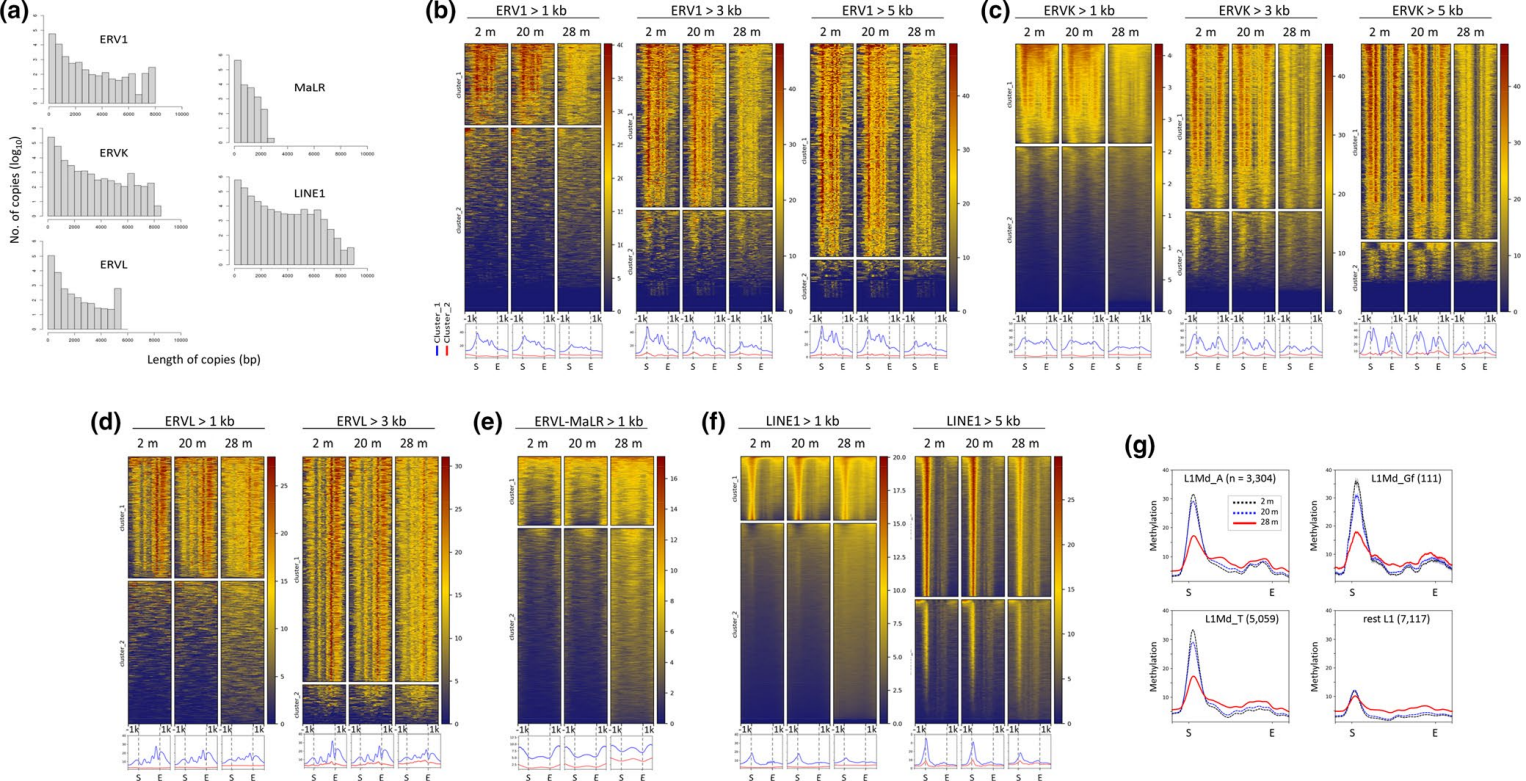
Global demethylation in large‐sized retroelements in the skeletal muscle of superaged mice. (a) Length distribution (in base pairs) of retroelement copies in each family. (b–f) Heatmaps of DNA methylation densities of ERV1 (b), ERVK (c), ERVL (d), ERVL‐MaLR (e), and LINE1 (f). Genomic copies of each retroelement family were subdivided by length into >1, >3, and >5 kb groups. Because of their shortness (<2.0 kb), MaLR family members only >1.0 kb in length were examined. Graphs at the bottom show the mean methylation levels of the two clusters (the blue line for cluster 1 and red line for cluster 2) separately. (g) The mean methylation levels of the evolutionarily active LINE1 families (L1Md_A, L1Md_Gf, and L1Md_T; >5 kb in size) and the rest of the LINE1 families (rest L1) in skeletal muscles of 2m (black), 20m (blue), and 28m (red) mice. The number of the members of each family is indicated in the parenthesis

Since the retroelement copies in the ERV and LINE1 families are present in diverse sizes (ranging from tens to thousands of base pairs) across the genome, we interrogated the length distribution of the retroelements across the genome. The sizes of the full‐length ERVs (except ERVL‐MaLR) and LINE1 vary and are approximately 5–7 kb on average (Crichton, Dunican, Maclennan, Meehan, & Adams, 2014). However, the majority of LINE1 and ERV elements in the genome are shorter than 1 kb (Figure 3a), indicating that the short copies were predominant in the methylation heatmap shown in Figure 2 and Figure S2. This finding prompted us to reanalyze those retroelements by size and to search for a size‐dependent manifestation of demethylation in relation to the functionality of large retroelements. We subdivided the genomic copies of each retroelement family into three groups as follows: those >1, >3, and >5 kb. As illustrated in Figure 3b–f, unique methylation patterns were unearthed in the methylation heatmaps of large retroelement fractions, with alternating peaks and valleys of DNA methylation along the retroelement bodies, particularly around the start and end loci. Furthermore, we observed that these sharp methylation patterns became dull and blunt in 28‐m skeletal muscle by LOM, unambiguously in cluster 1 of the ERV1, ERVK, and LINE1 families and moderately in ERVLs. In contrast, cluster 2 of ERVL and LINE1s showed GOM. ERVL‐MaLR copies displayed GOM events only. Some LINE1 families including L1Md_A, L1Md_Gf, and L1Md_Tf are evolutionarily active and capable of independent mobilization with full‐length transcripts (Hardies et al., 2000; Sookdeo, Hepp, McClure, & Boissinot, 2013). When we inspected age‐related methylation dynamics in these three LINE1 families, we found that both the initial methylation level and the degree of LOM were far more prominent in these families than the rest LINE1s (Figure 3g, see also Figure S3). It raises a possibility that the age‐associated LINE1 demethylation can practically affect the genome stability in skeletal muscle through expression and transposition. We confirmed the age‐related demethylation at these retroelement loci in an independent MBD‐seq analysis (Figure S4). T cells exhibited the same methylation patterns, which suggests the preservation of the methylation patterns of retroelement families across the cell types, although T cells did not show any recognizable changes in their DNA methylation in superaged mice (Figure S5); these results agree with previous results that LINE1 methylation did not vary with age in human blood samples (Bollati et al., 2009; Talens et al., 2012).

**Figure 4:**
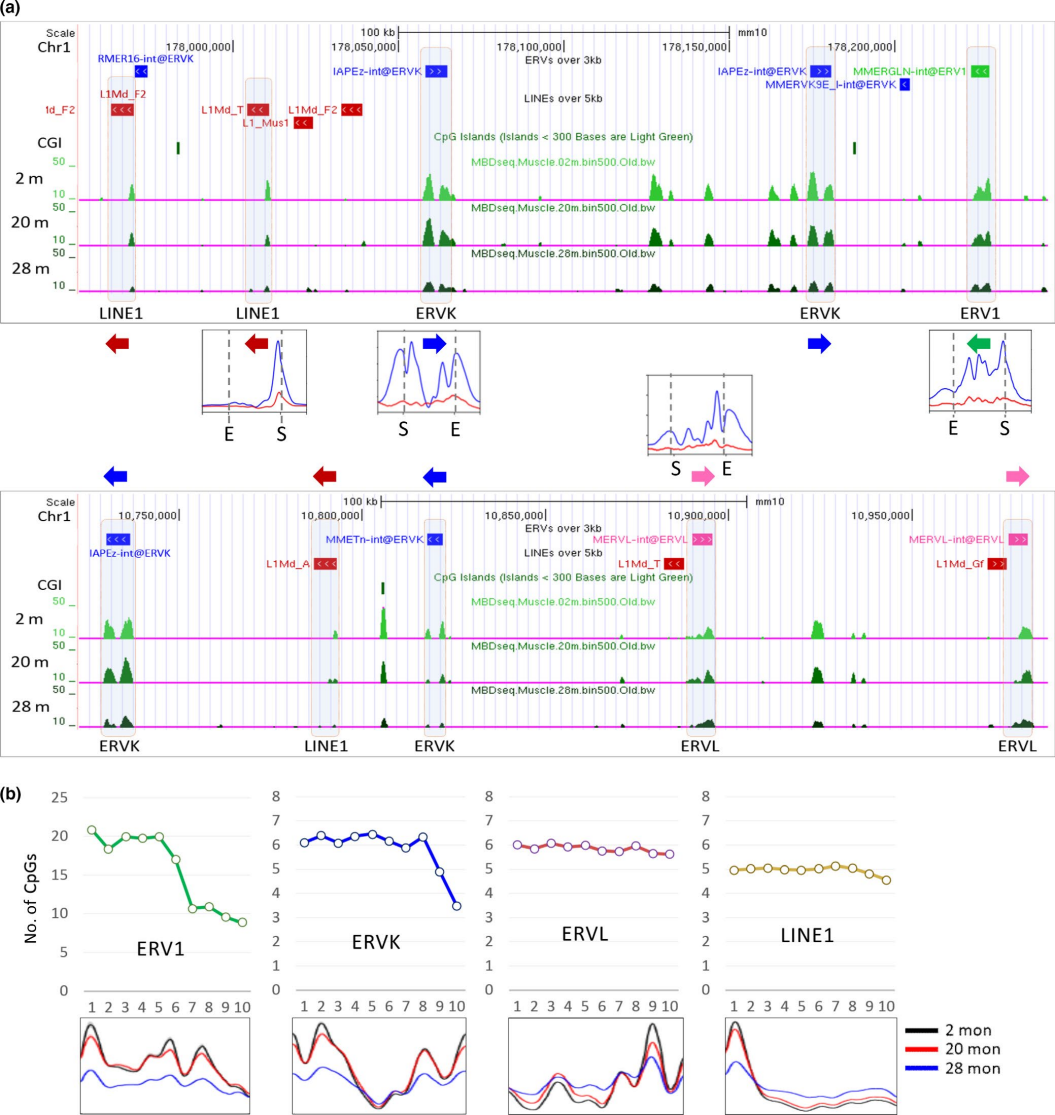
Specific methylation pattern in each retroelement family. (a) Distinctive patterns of DNA methylation among the various retroelement families. The genome browser shows only the large LINE1 (red boxes; >5.0 kb) and ERV copies (>3.0 kb; green for ERV1, blue for ERVK, and pink for ERVL) extracted from the repeatMasker (mm10) bed track. Arrows indicate the direction of transcription of the corresponding retroelements. The peak profiles of DNA methylation are borrowed from the results in Figure 2. (b) The relation of the observed methylation levels with the pattern of density of CpG dinucleotides within retroelements, which are subdivided into 10 equal parts

The heatmap of DNA methylation demonstrated that the retroelements had distinct methylation patterns of their own: Methylation peaks were found to be (a) strong in the 5′ long terminal repeat (LTR) and weak at the 3′ LTR in ERV1s; (b) weak at the 5′ LTR and strong at the 3′ LTR in ERVLs; (c) strong at both ends in ERVKs with the 5′ peak usually the stronger; and (d) as single distinguished peaks in the 5′ untranslated region (UTR) in LINE1s. To test whether these unique methylation patterns indeed manifested themselves from individual copies of retroelements, we extracted only the large LINE1 (>5.0 kb) and ERV (>3.0 kb) copies from the repeatMasker (mm10) track and uploaded them to the UCSC Genome Browser. The Genome Browser view in Figure 4a proved that the respective retroelements faithfully followed the specific patterns of their own families identified in the heatmap profiles with the peak heights lower in the 28‐m muscles than the peak heights in the younger muscles. The T cells manifested the same methylation patterns across the retroelements, but an age‐associated change in DNA methylation was not observed (Figure S6). Meanwhile, it was unlikely that the methylation peak simply mirrored the distribution of CpG sites along the retroelements because the CpG dinucleotides were relatively evenly dispersed within each retroelement (ERVK, ERVL, and LINE1; Figure 4b). One exception was the ERV1 family, in which the CpGs were the most abundant and biased to the 5′ half region, and this CpG distribution resembled the peak positions in the methylation profile of ERV1s.

### 2.5 Two different phases of retroelement expression during skeletal muscle aging

Since DNA demethylation at large retroelement sequences is related to the transcriptional derepression of the retroelements in superaged muscles, we investigated the genome‐wide retroelement expression. To estimate the expression levels of the retroelements, RNA‐seq was performed with the same batches of muscle tissues that were used for the methylation analysis (Table S1). The count distributions and the total numbers of genes expressed were similar among the samples, and principal component analysis separated the samples into groups of old and young skeletal muscles (Figure S7). To test the pertinence of the RNA‐seq data, we examined *Myh7*, the gene of type 1 (slow‐twitch) muscle fibers that is well known for its elevated expression in aged muscle (Ohlendieck, 2011); we also examined *Tnni1, Myl12a, Myom1, Myh6*, and *Actn2*, which are coexpressed with *Myh7* (STRING; https://string-db.org). Indeed, they were all significantly overrepresented in aged (20 m and 28 m) muscles (Figure S8a). In contrast, the gene expression levels of *Myh4*, type 2B (fast‐twitch) muscle fiber, and the coexpression genes, such as *Tnni2* and *Calm2*, were not different among the age groups (Figure S8b). Myod1, which plays a major role in muscle differentiation (Rudnicki et al., 1993), was significantly underrepresented in old muscles (Figure S8c), as was demonstrated in a previous study (Tamaki et al., 2000). Therefore, we concluded that our RNA‐seq data matched the known age‐related characteristics of skeletal muscle and were reliable for the age‐associated expression analysis of retroelements. For reference, the transcriptomic differences among the ages were highlighted by the fractions of differentially expressed genes (DEGs; fold change >2.0 and *p* < .05) in each age group (Figure S8d); by the number of DEGs detected, the transcriptomic difference was greater between the 2‐m and 20‐m muscles (*n* = 1,051) than it was between the 20‐m and 28‐m muscles (*n* = 348).

**Figure 5:**
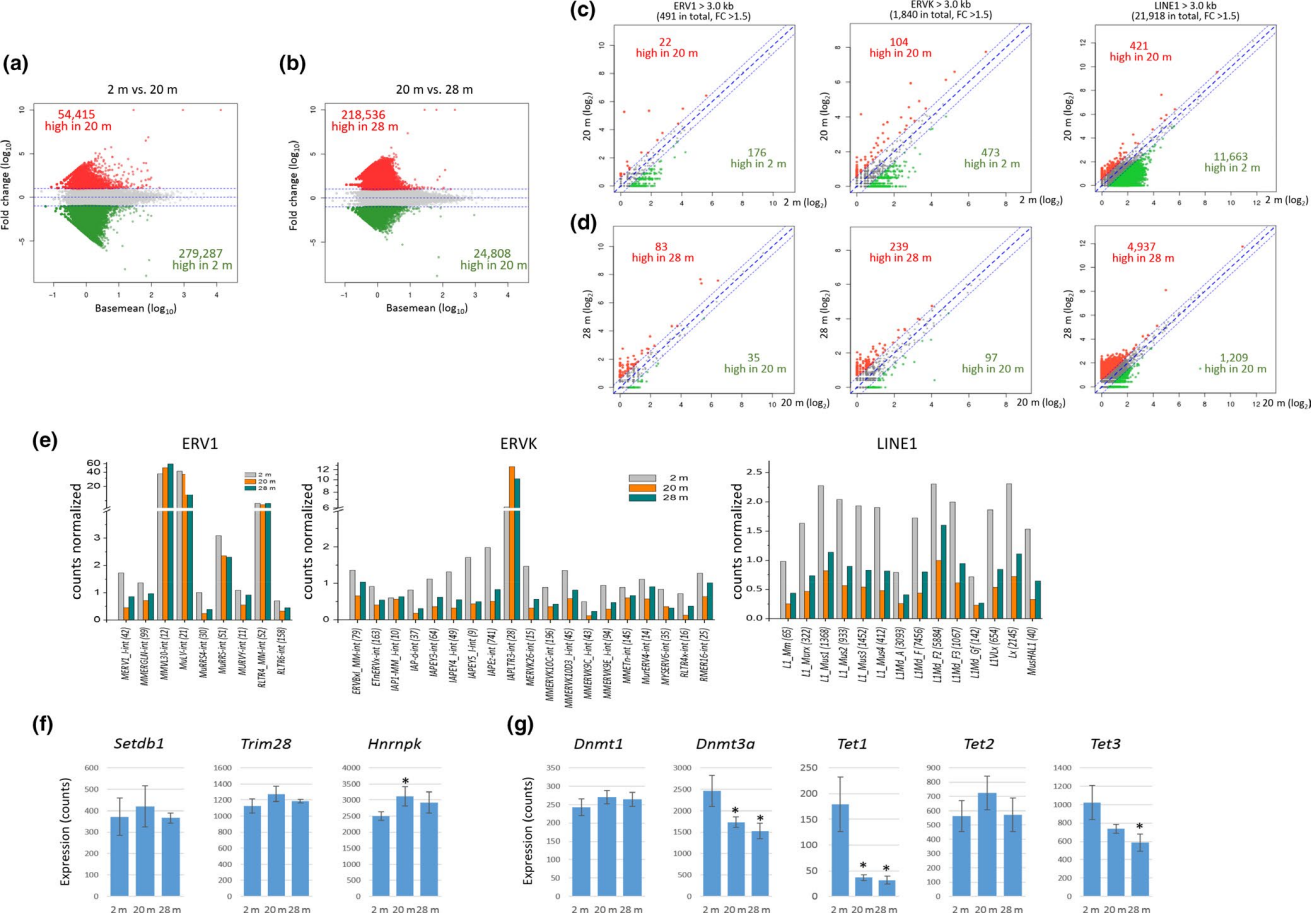
Changes in expression levels of retroelements during skeletal muscle aging. (a & b) MA plots for comparing the transcriptomes of whole retroelements between 2‐m and 20‐m muscles (a) or between 20‐m and 28‐m muscles (b). Transcriptome data were obtained through RNA‐seq with the skeletal muscles of the same mouse groups (2‐, 20‐, and 28‐month‐old female mice; *n* = 3 each group) as used in the methylation analysis, and as a result, ~26 million mapped reads (including multiple mapped reads) on average per sample with 91% mapping efficiency were obtained. The expression levels of retroelements were determined by employing the “repeatMasker” track downloaded from the UCSC Genome Browser. (c & d) Scatter plots for comparing the expression levels of large retroelements between 2‐m and 20‐m muscles (c) or between 20‐m and 28‐m muscles (d). In panels (a–d), the numbers of differentially expressed retroelements are indicated by different colors (green for younger samples and red for older ones). (e) Age‐associated changes in the transcript levels of various ERV1, ERVK, and LINE1 subfamilies. (f & g) Expression levels of genes involved in the repression of retroelements (f) and in the regulation of DNA methylation (g). Asterisks indicate significant differences (*p* < .05, Student's *t* test) from the levels in 2‐m muscles. Error bars: standard deviation

We examined the expression levels of retroelements, particularly those from the ERV1, ERVK, and LINE1 families, which showed obvious demethylation in superaged muscles. The differential expression analysis suggested that the retroelement expression markedly diminished in the 20‐m muscles compared to that in the young muscles (Figure 5a), and the reduced expression was more pronounced in the 28‐m muscles (Figure 5b). The same was true for large (>3 kb) copies of ERV1s, ERVKs, and LINE1s (Figure 5c,d); the expression levels of which were assessed by the number of reads uniquely mapped to the corresponding retroelement sequence in the genome using “hisat2.” When the expression levels of the ERV and LINE1 subfamilies were examined separately, most of them followed this fluctuating trend (Figure 5e). A similar result was obtained by using “SalmonTE” program, which measures the expression levels of transposable elements by family unit (Figure S9). We validated the expression of randomly chosen retroelements by quantitative real‐time PCR and indeed observed a similar trend in the retroelement expression levels in skeletal muscle with age (Figure S10). In summary, the results suggest that there are two stages of retroelement expression in skeletal muscles during aging: the global downregulation of retroelements in the early phase of aging and their derepression in the late phase of aging. The global increase in retroelement expression levels during the late phase of aging concurs with DNA demethylation at their loci in the skeletal muscles of superaged mice.

### 2.6 Expression patterns of the epi‐driver genes that potentially affect the epigenetic states of retroelements

To find the cause of the age‐associated fluctuation of retroelement expression, we analyzed the genes that are involved in the regulation of retroelements. Retroelement families are known to be repressed by the histone methyltransferase SETDB1 in cooperation with TRIM28 and HNRNPK (Matsui et al., 2010). As depicted in Figure 5f, the mean expression levels of the genes *Setdb1, Trim28*, and *Hnrnpk* also fluctuated in a pattern that was well matched to that of retroelement expression because they increased in the 20‐m muscles and then diminished in the 28‐m muscles. Nevertheless, the extent of the change in the respective genes was not very significant, except for Hnrnpk; this indicates that these genes may not be the cause of the fluctuation of the retroelement expression levels (see Discussion). Other genes, such as *G9a‐Glp* (Maksakova et al., 2013), *Suv39h1* (Bulut‐Karslioglu et al., 2014)*,* and *Ezh2* (Leeb et al., 2010), whose products are also known to repress retroelements transcriptionally, were not expressed differently between the young and older muscles (data not shown). In addition, we examined the expression levels of genes that function in the establishment, maintenance, and modification/erasure of CpG methylation to test the possibility that the abrupt demethylation in the retroelements was associated with the perturbed expression in this group of genes. The RNA‐seq data indicated that DNMT1 and DNMT3A are the DNA methyltransferase enzymes that primarily function in skeletal muscles, while DNMT3B and DNMT3L were barely expressed. Dnmt1 expression did not greatly change in aged muscles, whereas Dnmt3a expression significantly diminished (Figure 5g). The reduced DNMT3A level in the skeletal muscles may be the cause of global demethylation, but it cannot account for the observed methylation noise (i.e. a small and random increase in DNA methylation) across the genome. In addition, in the ten‐eleven translocation (TET) protein family, which is the newest group of DNA methylation “editors” and retroelement regulators (de la Rica et al., 2016), *Tet1* and *Tet3* were significantly downregulated in the 28 m muscles; however, *Tet2* was stably expressed. The connection of the reduced expression levels of the *Tet* genes with aging and DNA methylation changes in skeletal muscles is currently unknown (Tan & Shi, 2012).

## 3 DISCUSSION

We found that both the GOM (hypermethylation) and LOM (hypomethylation) events coexisted among the related members of each repeat family (ERV1, ERVK, LINE1, and SINE B1, B2, and B4) in superaged skeletal muscles (Figures 2 and 3). The discovery of the two contrasting patterns of methylation changes was attributed to the use of analytical tools, such as methylation‐based clustering and the size‐based fractionation of retroelement copies; this was believed because the analysis results with whole retroelement copies of each family produced just a simple one‐way change (Figure S2). This bidirectional pattern of methylation drift indicates that the age‐associated methylomic change may be driven by composite, antithetical forces instead of by a unidirectional flow of event (i.e., hypomethylation) that has been generally thought to sweep over the genomic repeats in aged cells. Following this “regression toward the mean” tendency of methylation drift, aging may tend to progressively reduce the preexisting methylation differences between genomic regions, the pattern of which had been established during muscle development and differentiation.

The gradual expansion of a disorder in the epigenomic structure leads to a perturbation of transcriptomic homeostasis and a steep rise of transcriptional noise; the latter is another landmark of aging and is a primary cause of variability in the gene expression between cells in an isogenic population (Enge et al., 2017). Supposing that DNA methylation is the primary mechanism regulating the transcription of genes in the corresponding regions, the LOM events in regions with heavy methylation may make the promoters more accessible to transcriptional activators, whereas the GOM events in the hypomethylated regions may make the promoters more and more occupied by repressors. This condition most likely results in a bimodal transcriptional drift, where the genes that are weakly expressed in the young animals are increasingly transcribed in older animals; in contrast, the genes that are highly expressed in the young animals are gradually downregulated with age (Min, Park, Jeon, et al., 2018). Our observation of age‐associated epigenetic drift therefore lends sound support to the bimodal transcriptional drift theory and is consistent with the general view that aging reflects a stochastic process of increasing disorder. Meanwhile, some studies observed only a weak correlation between DNA methylation (particularly at the promoters) and gene expression changes (Marttila et al., 2015; Slieker, Relton, Gaunt, Slagboom, & Heijmans, 2018; Steegenga et al., 2014). Notably, these studies used the Infinium 450K platform, which enables the identification of DNA methylation changes with a single CpG resolution (Bibikova et al., 2011). The base‐level sensitivity notwithstanding, it is unknown how well the selected few CpGs represent the surrounding cluster(s) of CpGs for the overall methylation state and whether the selection is grounded with an experimental basis (i.e., the connection with gene expression). Therefore, there is some difficulty directly linking the methylation of the selected CpGs to the expression of associated genes, and we assume that the difference between their studies and the present study lies in our approach to such a whole‐genome methylation analysis as MBD‐seq. As a drawback of the MBD‐seq method, it is semiquantitative in the sense that it does not yield direct estimates of CpG methylation levels. Thus, the previously suggested pipeline of methylome study (Aberg et al., 2012) looks relevant: MBD‐seq offers a first pass at global methylation analysis, and then, the identified genomic loci of interest are subject to a base‐level validation using technologies, such as pyrosequencing and targeted bisulfite PCR sequencing (TBP‐seq; Jeon, Min, Park, & Kang, 2017).

DNA methylation contributes to the transcriptional repression of retroelements, including the intracisternal A particles (IAPs) of the ERVK family. For example, IAP transcript levels are elevated 50‐ to 100‐fold in murine Dnmt1 knockout embryos, with a parallel demethylation of the IAP promoter (Walsh, Chaillet, & Bestor, 1998). Intracisternal A particles derepression occurs in stem cells of the male germline at undifferentiated stages when genome‐wide demethylation occurs. In addition, the induction of IAP expression occurs after treatment with demethylating agents, such as 5‐azacytidine (Hojman‐Montes de Oca et al., 1984). Retroelements, including Alu and LINE1s, were subject to derepression in cultured cells during senescence (De Cecco et al., 2013). As a possible mechanism for the derepression of retroelements, the authors of that study suggested the acquisition of a permissive chromatin state in senescent cells, which is likely related to a demethylation‐induced perturbation of the heterochromatin structure at retroelement loci (Sedivy, Banumathy, & Adams, 2008), as we have demonstrated in this study. Based on these precedent findings, the derepression of various retroelements could be predictable in superaged skeletal muscle, as we observed demethylation at various retroelements (Figure 3). In reality, the global demethylation event temporally coincides with an increase in the expression of retroelements, including the IAP sequences in skeletal muscles, as the mice advance in age from 20 to 28 months (Figure 5). Recent papers show that LINE1 expressions increase with age in mouse skeletal muscle (De Cecco et al., 2019; Simon et al., 2019), and our result on the age‐associated demethylation of LINE1, especially of those evolutionarily active LINE1 families (Figure 3g), now provides a mechanistic link with these observed increase in LINE1 expression. Meanwhile, the accumulation of retroelement‐derived transcripts and their reverse‐transcribed products in skeletal muscles may trigger an innate immune response and chronic inflammation through inducing IFN‐α and IFN‐β gene expressions, as suggested before (Bourque et al., 2018; De Cecco et al., 2019; Kang, 2018; Simon et al., 2019).

Various retroelements were more repressed in the 20‐m skeletal muscle than they were in the 2‐m muscle; then, these retroelements became derepressed in superaged muscle (Figure 5e). The tighter repression of retroelements in the 20‐m skeletal muscle appears to be independent of DNA methylation because there was no apparent methylation change in the retroelements during that time. It would be interesting to determine by what mechanism the retroelements become even further repressed during early aging and what the significance/purpose is in it. In addition to DNA methylation, histone methylation contributes to the repression of genomic retroelements. Retroelements are controlled by histone H3 lysine 9 methylation (H3K9me) catalyzed by SETDB1 [see (Kang, 2018) and references therein], G9A/GLP (Maksakova et al., 2013), and SUV39H1 and SUV39H2 (Bulut‐Karslioglu et al., 2014), and/or by H3 lysine 27 methylation (H3K27me) performed by the PRC2 complex involving EZH2 (Leeb et al., 2010). However, a recent study (Min, Park, Jeon, et al., 2018) has suggested that the expression levels of epi‐driver genes encoding these histone‐modifying enzymes and other related epigenetic modifiers were relatively stable and had little change during skeletal muscle aging. One seemingly tiny, but nonetheless noticeable, change was that the expression levels of the genes encoding members of the well‐known KRAB–ZNF–TRIM28–SETDB1–HNRNPK complex, which represses retroelements, all consistently manifested a negative correlation with the retroelement expression levels (Figure 5f). The same was true for TET2, which was recently reported to silence retroelements by the post‐transcriptional destabilization of their transcripts (Guallar et al., 2018). In this regard, we speculate that although the changes in the expression of the respective genes cannot lead to a significant outcome, the sum of their small changes can make a visible difference in the transcript levels of retroelements.

Skeletal muscles are made of different types of muscle fibers, and the composition changes with age (Ohlendieck, 2011). Given that one of the major drivers of DNA methylation pattern is cell type, it could be that the altered methylation pattern in the superaged muscle reflects a change in the composition of muscle fiber types with age. We only know that the human type 1 slow‐twitch and type 2a fast‐twitch muscle fibers had similar levels and distributions of methylated CpGs at promoters and CGIs (Begue, Raue, Jemiolo, & Trappe, 2017). However, there is no information about whether the different muscle fibers undergo unique methylation changes in their own pattern with age. In this study, we noticed that the type 1 slow‐twitch muscle fiber genes, such as *Myh7, Tnni1, Myl12a, Myom1, Myh6*, and *Actn2*, which are expressed at higher levels in aged muscle (Ohlendieck, 2011), were increasingly expressed in the 20‐m and 28‐m muscles compared to their expression in the 2‐m muscles (seen in Figure S8). However, given no further difference between the 20‐m and 28‐m muscles, we hypothesize that the muscle fiber composition is not very different between the 20‐m and 28‐m muscles and, accordingly, that the distinct methylation pattern in superaged muscle is ascribable to the aging process itself, rather than to the age‐associated change in muscle composition. Nevertheless, we cannot completely exclude the possibility of age‐specific compositional changes in other types of muscle cells that can make differences in the DNA methylation patterns in aged skeletal muscles. If this were the case, we could tell that the regression to the mean tendency in skeletal muscles is rooted in the presence of the variety of cell types and that the lack of such a tendency in the MAX‐purified T cells appears to make sense.

The degree of DNA methylation in retroelements can affect the expression levels of the associated genes. A well‐known example is the agouti viable yellow (*A^vy^*) allele. The *A^vy^* allele contains an IAP insertion close to the transcription start site of the agouti gene, and its expression level inversely correlates with the CpG methylation state of the IAP sequence (Michaud et al., 1994). In *Arabidopsis thaliana*, the methylation status of intronic transposable elements was observed to correlate with lower transcription levels of the associated genes with TE insertions (Le, Miyazaki, Takuno, & Saze, 2015). In this study, we observed two categories of large retroelements: the heavily methylated copies being demethylated with aging and the other, poorly methylated copies (cluster 2), which constitute approximately 10%–30% in each retroelement family and naturally do not undergo a methylation change (Figure 3b–3f). Thus, those genes that are intimately associated with the former group of retroelements may be transcriptionally modulated after the demethylation of retroelements during aging. If so, these genes are considered age‐sensitive and can serve as marker genes for skeletal muscle aging.

## 4 EXPERIMENTAL PROCEDURES

### 4.1 Isolation of splenic T cells and skeletal muscles

To obtain skeletal muscles and T cells, C57BL/6J female mice at 2 months (*n* = 3), 20 months (*n* = 3), and 28 months (*n* = 3) of ages were sacrificed, and their spleens and skeletal muscles in hindlimbs were surgically removed. Mice about 28 months of age, which correlates with humans ranging from 76 to 78 years of age, can be considered “very old” and generally show a 50% survivorship (The Jackson Laboratory; lifespan as a biomarker). For isolation of T cells, a spleen from each mouse was ground with frosted glass slides followed by a series of syringe homogenization as shrinking the needle sizes (from 18 to 27 gauges), and T cells were isolated by magnetic‐activated cell sorting (MACS; Pan T Cell Isolation Kit II, mouse; Miltenyi Biotec) as described elsewhere (Park et al., 2017). For skeletal muscles, the whole muscle tissues were completely frozen in liquid nitrogen, and they were ground to powder using a mortar and a pestle. The powdered tissues were further homogenized using a Biomasher II (DWK Life Sciences) in tissue lysis buffer (ATL buffer; Qiagen).

### 4.2 Construction of MBD‐seq libraries

Genomic DNAs (gDNAs) were extracted from the tissue samples using DNeasy Blood & Tissue Kit (Qiagen) according to the manufacturer's instruction. Genomic DNAs obtained from three mice of the same ages were pooled before sonication using Bioruptor Pico (Diagenode) and purified using with AMPure XP beads (Beckman) (Min, Park, & Kang, 2018). For preparation of MBD capture, 7 μl MBD‐Biotin proteins per sample from MethylMiner Methylated DNA Enrichment Kit (Thermo) was first incubated with 10 μl of precleared Dynabeads (Thermo) at RT for 1 hr. Methylated DNAs were captured by incubating 1 μg of sonicated gDNA fragments with MBD beads at 4°C for overnight. Beads were washed twice, and captured DNA fragments were eluted using 2 M NaCl and precipitated using ethanol. Enrichment of methylated DNA fragments was checked by PCR for control sets of methylated and nonmethylated DNAs (Thermo) that were premixed as spike‐ins. Using the enriched methylated DNA fragments, Illumina sequencing libraries were generated. All the captured DNAs were end‐repaired by reacting with T4 DNA polymerase (NEB), Klenow (NEB), and T4 polynucleotide kinase (NEB) in the presence of dNTP at 20°C for 2 hr, and then 3′‐end adenylated using Klenow fragment (NEB) at 30°C for 2 hr. Finally, the end‐repaired DNA fragments were ligated with NEBNext adapters (NEB) and were enriched by 18 cycles of PCR. The resulting libraries were subjected to Illumina sequencing with NEXTseq 500. To confirm the MBD‐seq result, we repeated the same experiment of MBD capture and sequencing using the same set of pooled gDNAs as in the first‐round MBD‐seq experiment.

### 4.3 RNA‐seq library construction

Three biological replicates for each age group were used in the RNA‐seq analysis. Thirty mg of the powdered muscle tissues in liquid nitrogen was homogenized and lysed with 200 μl of tissue isolation buffer (RTL) of RNeasy Plus Mini Kit (QIAGEN) in Biomasher II (DWK Life Sciences). Total RNAs were obtained from the same animals as used in the MBD‐seq analysis and treated with DNase I. Poly‐A tailed RNAs were isolated from 1 μg of total RNAs using Dynabeads mRNA DIRECT Kit (Thermo) and using First‐Strand Buffer (NEB) in the library construction kit, the captured RNAs were eluted. RNA‐seq libraries were constructed using NEBNext Ultra RNA Library Prep Kit for Illumina (NEB). Isolated poly‐A tailed RNAs were incubated at 94°C for 15 min for fragmentation. First‐strand cDNA was synthesized with fragmented RNAs using ProtoScript II Reverse Transcriptase, and their second strands were synthesized using Second‐Strand Synthesis Enzyme Mix in the kit before purification. After the end repair of the double‐stranded DNAs using NEBNext End Prep Enzyme Mix in the condition of 20°C for 30 m and 65°C for 30 m, the products were incubated with NEBNext Adaptor and Blunt/TA Ligase Master Mix (NEB) at 20°C for 15 m. The resulting ligates were enriched by 13 cycles of PCR using NEBNext Q5 Hot Start HiFi PCR Master Mix and index primers. Enriched RNA‐seq libraries were purified and sequenced using NextSeq 500 (Illumina).

### 4.4 Bioinformatic analyses of MBD‐seq and RNA‐seq results

Raw sequencing reads in both MBD‐seq and RNA‐seq were preprocessed to remove adapter sequences and low‐quality bases using “Trim_galor” (https://github.com/FelixKrueger/TrimGalore/archive/0.4.5.zip) with the default setting for paired‐end trimming. For analysis of MBD‐seq results, the trimmed reads were mapped on the mouse genome (mm10) using Bowtie2 with default options. MBD‐seq enrichment in FPKM over the genomic intervals was estimated using “bamCoverage” in deepTools. Read coverages over retroelements were calculated by “computeMatrix” with the repeat masker track from UCSC genome browser, and heatmaps and coverage plots were generated using the count matrix by “plotHeatmap” and “plotProfile,” respectively. The URL for the UCSC custom track is https://genome.ucsc.edu/cgi-bin/hgTracks?db=mm10&lastVirtModeType=default&lastVirtModeExtraState=&virtModeType=default&virtMode=0&nonVirtPosition=&position=chr1%3A128667231%2D132278505&hgsxml:id=702446709_DAKxzdnCB1ZHjs9MoLrHujtP6A0L. To identify the differentially methylated regions (DMRs), reads fell in 500 bases of genomic bins were counted using “makewindows” and “intersect” in bedtools (https://bedtools.readthedocs.io/en/latest/). The count data of individual samples were merged, and normalization and differential methylation analysis was performed using DESeq2.

For analysis of RNA‐seq results, the trimmed reads were aligned on the reference genome (mm10) using Hisat2 with “‐‐dta” option for splice junction mapping. For estimation of gene/retroelement expression, “refSeq” and “repeatMasker” tracks, respectively, were downloaded from UCSC genome browser in GTF, and based on the genomic coordination in GTF files, reads were counted using “htseq‐count” with “intersection‐strict” parameter. All count data were merged into one data table using a home‐brew bash script, and the raw count data were normalized and used for differential expression analysis with “DESeq2.” Genes or retroelements with fold change >2 and *p*‐value <.05 were considered differential expression. Bam files were converted to bigWig files for visualization on UCSC genome browser using “bamCoverage” in “deepTools.”

### 4.5 Validation—retroelement expression

Total RNAs were extracted from the muscle tissue of each age group as described above. Reverse transcription was performed by incubating 1 μg of DNase I‐pretreated RNA with Superscript III enzyme (Invitrogen), 20 μM oligo‐dT primers (Invitrogen), and 50 ng random hexamers (Invitrogen) at 50°C for 1 hr. Ten ng of the synthesized cDNAs was used for real‐time quantitative PCR (QuantStudio3 Real‐Time PCR system, ABI) with the specific primers for individual retroelement subfamilies (Table S1). The PCR was performed with a following program, 10 min of predenaturation at 95°C followed by 40 cycles of 95°C for 15 s, 55°C for 15 s, and 72°C for 1 m. Finally, relative expression levels of each retroelement against *Gapdh* were calculated using QuantStudio Design & Analysis Software (Thermo).

## CONFLICT OF INTEREST

The authors have no conflict of interest to declare.

## AUTHORS' CONTRIBUTION

BM conducted bioinformatic analyses and constructed MBD‐seq libraries. KJ constructed RNA‐seq libraries. JSP maintained and provided mice. YKK designed and supervised the study, performed bioinformatic analyses, and wrote the manuscript.

## Data Availability

The data that support the findings of this study are openly available in Gene Expression Omnibus (GEO) with accession number of GSE123954.

## References

[acel13042-bib-0001] Aberg, K. A. , McClay, J. L. , Nerella, S. , Xie, L. Y. , Clark, S. L. , Hudson, A. D. , … van den Oord, E. J. (2012). MBD‐seq as a cost‐effective approach for methylome‐wide association studies: Demonstration in 1500 case–control samples. Epigenomics, 4, 605–621. 10.2217/epi.12.59 23244307 PMC3923085

[acel13042-bib-0002] Begue, G. , Raue, U. , Jemiolo, B. , & Trappe, S. (2017). DNA methylation assessment from human slow‐ and fast‐twitch skeletal muscle fibers. Journal of Applied Physiology, 122(4), 952–967. 10.1152/japplphysiol.00867.2016 28057818 PMC5407195

[acel13042-bib-0003] Bibikova, M. , Barnes, B. , Tsan, C. , Ho, V. , Klotzle, B. , Le, J. M. , … Shen, R. (2011). High density DNA methylation array with single CpG site resolution. Genomics, 98, 288–295. 10.1016/j.ygeno.2011.07.007 21839163

[acel13042-bib-0004] Bjornsson, H. T. , Sigurdsson, M. I. , Fallin, M. D. , Irizarry, R. A. , Aspelund, T. , Cui, H. , … Feinberg, A. P. (2008). Intra‐individual change over time in DNA methylation with familial clustering. JAMA, 299, 2877–2883. 10.1001/jama.299.24.2877 18577732 PMC2581898

[acel13042-bib-0005] Bollati, V. , Schwartz, J. , Wright, R. , Litonjua, A. , Tarantini, L. , Suh, H. , … Baccarelli, A. (2009). Decline in genomic DNA methylation through aging in a cohort of elderly subjects. Mechanisms of Ageing and Development, 130, 234–239. 10.1016/j.mad.2008.12.003 19150625 PMC2956267

[acel13042-bib-0006] Bormann, F. , Rodriguez‐Paredes, M. , Hagemann, S. , Manchanda, H. , Kristof, B. , Gutekunst, J. , … Lyko, F. (2016). Reduced DNA methylation patterning and transcriptional connectivity define human skin aging. Aging Cell, 15, 563–571. 10.1111/acel.12470 27004597 PMC4854925

[acel13042-bib-0007] Bourque, G. , Burns, K. H. , Gehring, M. , Gorbunova, V. , Seluanov, A. , Hammell, M. , … Feschotte, C. (2018). Ten things you should know about transposable elements. Genome Biology, 19, 199 10.1186/s13059-018-1577-z 30454069 PMC6240941

[acel13042-bib-0008] Brunk, B. P. , Goldhamer, D. J. , & Emerson, C. P. Jr (1996). Regulated demethylation of the myoD distal enhancer during skeletal myogenesis. Developmental Biology, 177, 490–503. 10.1006/dbio.1996.0180 8806826

[acel13042-bib-0009] Bulut‐Karslioglu, A. , De La Rosa‐Velazquez, I. A. , Ramirez, F. , Barenboim, M. , Onishi‐Seebacher, M. , Arand, J. , … Jenuwein, T. (2014). Suv39h‐dependent H3K9me3 marks intact retrotransposons and silences LINE elements in mouse embryonic stem cells. Molecular Cell, 55, 277–290. 10.1016/j.molcel.2014.05.029 24981170

[acel13042-bib-0010] Carrio, E. , & Suelves, M. (2015). DNA methylation dynamics in muscle development and disease. Frontiers in Aging Neuroscience, 7, 19 10.3389/fnagi.2015.00019 25798107 PMC4350440

[acel13042-bib-0011] Coelho Junior, H. J. , Gambassi, B. B. , Diniz, T. A. , Fernandes, I. M. , Caperuto, E. C. , Uchida, M. C. , … Rodrigues, B. (2016). Inflammatory mechanisms associated with skeletal muscle sequelae after stroke: Role of physical exercise. Mediators of Inflammation, 2016, 3957958 10.1155/2016/3957958 27647951 PMC5018330

[acel13042-bib-0012] Crichton, J. H. , Dunican, D. S. , Maclennan, M. , Meehan, R. R. , & Adams, I. R. (2014). Defending the genome from the enemy within: Mechanisms of retrotransposon suppression in the mouse germline. Cellular and Molecular Life Sciences, 71, 1581–1605. 10.1007/s00018-013-1468-0 24045705 PMC3983883

[acel13042-bib-0013] Cruz‐Jentoft, A. J. , Bahat, G. , Bauer, J. , Boirie, Y. , Bruyere, O. , Cederholm, T. , … Writing Group for the European Working Group on Sarcopenia in Older People 2 (EWGSOP2), and the Extended Group for EWGSOP2 (2019). Sarcopenia: Revised European consensus on definition and diagnosis. Age and Ageing, 48, 16–31. 10.1093/ageing/afy169 30312372 PMC6322506

[acel13042-bib-0014] Day, K. , Waite, L. L. , Thalacker‐Mercer, A. , West, A. , Bamman, M. M. , Brooks, J. D. , … Absher, D. (2013). Differential DNA methylation with age displays both common and dynamic features across human tissues that are influenced by CpG landscape. Genome Biology, 14, R102 10.1186/gb-2013-14-9-r102 24034465 PMC4053985

[acel13042-bib-0015] De Cecco, M. , Criscione, S. W. , Peckham, E. J. , Hillenmeyer, S. , Hamm, E. A. , Manivannan, J. , … Sedivy, J. M. (2013). Genomes of replicatively senescent cells undergo global epigenetic changes leading to gene silencing and activation of transposable elements. Aging Cell, 12, 247–256. 10.1111/acel.12047 23360310 PMC3618682

[acel13042-bib-0016] De Cecco, M. , Ito, T. , Petrashen, A. P. , Elias, A. E. , Skvir, N. J. , Criscione, S. W. , … Sedivy, J. M. (2019). L1 drives IFN in senescent cells and promotes age‐associated inflammation. Nature, 566, 73–78. 10.1038/s41586-018-0784-9 30728521 PMC6519963

[acel13042-bib-0017] de la Rica, L. , Deniz, O. , Cheng, K. C. , Todd, C. D. , Cruz, C. , Houseley, J. , & Branco, M. R. (2016). TET‐dependent regulation of retrotransposable elements in mouse embryonic stem cells. Genome Biology, 17, 234 10.1186/s13059-016-1096-8 27863519 PMC5116139

[acel13042-bib-0018] Enge, M. , Arda, H. E. , Mignardi, M. , Beausang, J. , Bottino, R. , Kim, S. K. , & Quake, S. R. (2017). Single‐cell analysis of human pancreas reveals transcriptional signatures of aging and somatic mutation patterns. Cell, 171(2), 321–330.e14. 10.1016/j.cell.2017.09.004 28965763 PMC6047899

[acel13042-bib-0019] Fuso, A. , Ferraguti, G. , Grandoni, F. , Ruggeri, R. , Scarpa, S. , Strom, R. , & Lucarelli, M. (2010). Early demethylation of non‐CpG, CpC‐rich, elements in the myogenin 5'‐flanking region: A priming effect on the spreading of active demethylation. Cell Cycle, 9, 3965–3976. 10.4161/cc.9.19.13193 20935518 PMC3047754

[acel13042-bib-0020] Guallar, D. , Bi, X. , Pardavila, J. A. , Huang, X. , Saenz, C. , Shi, X. , … Wang, J. (2018). RNA‐dependent chromatin targeting of TET2 for endogenous retrovirus control in pluripotent stem cells. Nature Genetics, 50, 443–451. 10.1038/s41588-018-0060-9 29483655 PMC5862756

[acel13042-bib-0021] Haithcock, E. , Dayani, Y. , Neufeld, E. , Zahand, A. J. , Feinstein, N. , Mattout, A. , … Liu, J. (2005). Age‐related changes of nuclear architecture in *Caenorhabditis elegans* . Proceedings of the National Academy of Sciences of the United States of America, 102, 16690–16695. 10.1073/pnas.0506955102 16269543 PMC1283819

[acel13042-bib-0022] Hardies, S. C. , Wang, L. , Zhou, L. , Zhao, Y. , Casavant, N. C. , & Huang, S. (2000). LINE‐1 (L1) lineages in the mouse. Molecular Biology and Evolution, 17, 616–628. 10.1093/oxfordjournals.molbev.a026340 10742052

[acel13042-bib-0023] Hojman‐Montes de Oca, F. , Lasneret, J. , Dianoux, L. , Canivet, M. , Ravicovitch‐Ravier, R. , & Peries, J. (1984). Regulation of intracisternal A particles in mouse teratocarcinoma cells: Involvement of DNA methylation in transcriptional control. Biology of the Cell, 52, 199–204. 6085276 10.1111/j.1768-322x.1985.tb00337.x

[acel13042-bib-0024] Horvath, S. (2013). DNA methylation age of human tissues and cell types. Genome Biology, 14, R115 10.1186/gb-2013-14-10-r115 24138928 PMC4015143

[acel13042-bib-0025] Hu, B. , Gharaee‐Kermani, M. , Wu, Z. , & Phan, S. H. (2010). Epigenetic regulation of myofibroblast differentiation by DNA methylation. American Journal of Pathology, 177, 21–28. 10.2353/ajpath.2010.090999 20489138 PMC2893647

[acel13042-bib-0026] Hughes, V. A. , Frontera, W. R. , Wood, M. , Evans, W. J. , Dallal, G. E. , Roubenoff, R. , & Fiatarone Singh, M. A. (2001). Longitudinal muscle strength changes in older adults: Influence of muscle mass, physical activity, and health. Journals of Gerontology. Series A, Biological Sciences and Medical Sciences, 56, B209–217. 10.1093/gerona/56.5.B209 11320101

[acel13042-bib-0027] Jeon, K. , Min, B. , Park, J. S. , & Kang, Y. K. (2017). Simultaneous methylation‐level assessment of hundreds of CpG sites by targeted bisulfite PCR sequencing (TBPseq). Frontiers in Genetics, 8, 97 10.3389/fgene.2017.00097 28751909 PMC5507944

[acel13042-bib-0028] Jin, L. , Jiang, Z. , Xia, Y. , Lou, P. , Chen, L. , Wang, H. , … Li, M. (2014). Genome‐wide DNA methylation changes in skeletal muscle between young and middle‐aged pigs. BMC Genomics, 15, 653 10.1186/1471-2164-15-653 25096499 PMC4147169

[acel13042-bib-0029] Jintaridth, P. , & Mutirangura, A. (2010). Distinctive patterns of age‐dependent hypomethylation in interspersed repetitive sequences. Physiological Genomics, 41, 194–200. 10.1152/physiolgenomics.00146.2009 20145203

[acel13042-bib-0030] Kang, Y. K. (2018). Surveillance of retroelement expression and nucleic‐acid immunity by histone methyltransferase SETDB1. BioEssays, 40(9), e1800058 10.1002/bies.201800058 29897144

[acel13042-bib-0031] Larson, K. , Yan, S. J. , Tsurumi, A. , Liu, J. , Zhou, J. , Gaur, K. , … Li, W. X. (2012). Heterochromatin formation promotes longevity and represses ribosomal RNA synthesis. PLoS Genetics, 8, e1002473 10.1371/journal.pgen.1002473 22291607 PMC3266895

[acel13042-bib-0032] Le, T. N. , Miyazaki, Y. , Takuno, S. , & Saze, H. (2015). Epigenetic regulation of intragenic transposable elements impacts gene transcription in *Arabidopsis thaliana* . Nucleic Acids Research, 43, 3911–3921. 25813042 10.1093/nar/gkv258 PMC4417168

[acel13042-bib-0033] Lee, E. , Iskow, R. , Yang, L. , Gokcumen, O. , Haseley, P. , Luquette, L. J. , … Cancer Genome Atlas Research Network (2012). Landscape of somatic retrotransposition in human cancers. Science, 337, 967–971. 10.1126/science.1222077 22745252 PMC3656569

[acel13042-bib-0034] Leeb, M. , Pasini, D. , Novatchkova, M. , Jaritz, M. , Helin, K. , & Wutz, A. (2010). Polycomb complexes act redundantly to repress genomic repeats and genes. Genes & Development, 24, 265–276. 10.1101/gad.544410 20123906 PMC2811828

[acel13042-bib-0035] Maksakova, I. A. , Thompson, P. J. , Goyal, P. , Jones, S. J. , Singh, P. B. , Karimi, M. M. , & Lorincz, M. C. (2013). Distinct roles of KAP1, HP1 and G9a/GLP in silencing of the two‐cell‐specific retrotransposon MERVL in mouse ES cells. Epigenetics Chromatin, 6, 15 10.1186/1756-8935-6-15 23735015 PMC3682905

[acel13042-bib-0036] Marttila, S. , Kananen, L. , Hayrynen, S. , Jylhava, J. , Nevalainen, T. , Hervonen, A. , … Hurme, M. (2015). Ageing‐associated changes in the human DNA methylome: Genomic locations and effects on gene expression. BMC Genomics, 16, 179 10.1186/s12864-015-1381-z 25888029 PMC4404609

[acel13042-bib-0037] Matsui, T. , Leung, D. , Miyashita, H. , Maksakova, I. A. , Miyachi, H. , Kimura, H. , … Shinkai, Y. (2010). Proviral silencing in embryonic stem cells requires the histone methyltransferase ESET. Nature, 464, 927–931. 10.1038/nature08858 20164836

[acel13042-bib-0038] Michaud, E. J. , van Vugt, M. J. , Bultman, S. J. , Sweet, H. O. , Davisson, M. T. , & Woychik, R. P. (1994). Differential expression of a new dominant agouti allele (Aiapy) is correlated with methylation state and is influenced by parental lineage. Genes & Development, 8, 1463–1472. 10.1101/gad.8.12.1463 7926745

[acel13042-bib-0039] Min, B. , Park, J. S. , & Kang, Y.‐K. (2018). Determination of oocyte‐manipulation, zygote‐manipulation, and genome‐reprogramming effects on the transcriptomes of bovine blastocysts. Frontiers in Genetics, 9,143 10.3389/fgene.2018.00143 29740477 PMC5928200

[acel13042-bib-0040] Min, B. , Park, M. , Jeon, K. , Park, J. S. , Seo, H. , Jeong, S. , & Kang, Y. K. (2018). Age‐associated bimodal transcriptional drift reduces intergenic disparities in transcription. Aging, 10, 789–807. 10.18632/aging.101428 29706608 PMC5940109

[acel13042-bib-0041] Montesano, A. , Luzi, L. , Senesi, P. , & Terruzzi, I. (2013). Modulation of cell cycle progression by 5‐azacytidine is associated with early myogenesis induction in murine myoblasts. International Journal of Biological Sciences, 9, 391–402. 10.7150/ijbs.4729 23678289 PMC3654436

[acel13042-bib-0042] Ohlendieck, K. (2011). Proteomic profiling of fast‐to‐slow muscle transitions during aging. Frontiers in Physiology, 2, 105 10.3389/fphys.2011.00105 22207852 PMC3245893

[acel13042-bib-0043] Pal, S. , & Tyler, J. K. (2016). Epigenetics and aging. Science Advances, 2, e1600584 10.1126/sciadv.1600584 27482540 PMC4966880

[acel13042-bib-0044] Park, M. , Min, B. , Jeon, K. , Cho, S. , Park, J. S. , Kim, J. , … Kang, Y. K. (2017). Age‐associated chromatin relaxation is enhanced in Huntington's disease mice. Aging, 9, 803–822. 10.18632/aging.101193 28288000 PMC5391233

[acel13042-bib-0045] Reilly, M. T. , Faulkner, G. J. , Dubnau, J. , Ponomarev, I. , & Gage, F. H. (2013). The role of transposable elements in health and diseases of the central nervous system. Journal of Neuroscience, 33, 17577–17586. 10.1523/JNEUROSCI.3369-13.2013 24198348 PMC3818539

[acel13042-bib-0046] Rudnicki, M. A. , Schnegelsberg, P. N. , Stead, R. H. , Braun, T. , Arnold, H. H. , & Jaenisch, R. (1993). MyoD or Myf‐5 is required for the formation of skeletal muscle. Cell, 75, 1351–1359. 10.1016/0092-8674(93)90621-V 8269513

[acel13042-bib-0047] Sedivy, J. M. , Banumathy, G. , & Adams, P. D. (2008). Aging by epigenetics–a consequence of chromatin damage? Experimental Cell Research, 314, 1909–1917. 18423606 10.1016/j.yexcr.2008.02.023 PMC2464300

[acel13042-bib-0048] Sedivy, J. M. , Kreiling, J. A. , Neretti, N. , De Cecco, M. , Criscione, S. W. , Hofmann, J. W. , … Peterson, A. L. (2013). Death by transposition – The enemy within? BioEssays, 35, 1035–1043. 24129940 10.1002/bies.201300097 PMC3922893

[acel13042-bib-0049] Simon, M. , Van Meter, M. , Ablaeva, J. , Ke, Z. , Gonzalez, R. S. , Taguchi, T. , … Gorbunova, V. (2019). LINE1 Derepression in aged wild‐type and SIRT6‐deficient mice drives inflammation. Cell Metabolism, 29(871–885), e875 10.1016/j.cmet.2019.02.014 PMC6449196 30853213

[acel13042-bib-0050] Slieker, R. C. , Relton, C. L. , Gaunt, T. R. , Slagboom, P. E. , & Heijmans, B. T. (2018). Age‐related DNA methylation changes are tissue‐specific with ELOVL2 promoter methylation as exception. Epigenetics Chromatin., 11, 25 10.1186/s13072-018-0191-3 29848354 PMC5975493

[acel13042-bib-0051] Sookdeo, A. , Hepp, C. M. , McClure, M. A. , & Boissinot, S. (2013). Revisiting the evolution of mouse LINE‐1 in the genomic era. Mobile DNA, 4, 3 10.1186/1759-8753-4-3 23286374 PMC3600994

[acel13042-bib-0052] Steegenga, W. T. , Boekschoten, M. V. , Lute, C. , Hooiveld, G. J. , de Groot, P. J. , Morris, T. J. , … Muller, M. (2014). Genome‐wide age‐related changes in DNA methylation and gene expression in human PBMCs. Age (Dordr), 36, 9648 10.1007/s11357-014-9648-x 24789080 PMC4082572

[acel13042-bib-0053] Szyf, M. , Rouleau, J. , Theberge, J. , & Bozovic, V. (1992). Induction of myogenic differentiation by an expression vector encoding the DNA methyltransferase cDNA sequence in the antisense orientation. Journal of Biological Chemistry, 267, 12831–12836. 1618783

[acel13042-bib-0054] Talens, R. P. , Christensen, K. , Putter, H. , Willemsen, G. , Christiansen, L. , Kremer, D. , … Heijmans, B. T. (2012). Epigenetic variation during the adult lifespan: Cross‐sectional and longitudinal data on monozygotic twin pairs. Aging Cell, 11, 694–703. 10.1111/j.1474-9726.2012.00835.x 22621408 PMC3399918

[acel13042-bib-0055] Tamaki, T. , Uchiyama, S. , Uchiyama, Y. , Akatsuka, A. , Yoshimura, S. , Roy, R. R. , & Edgerton, V. R. (2000). Limited myogenic response to a single bout of weight‐lifting exercise in old rats. American Journal of Physiology. Cell Physiology, 278, C1143–C1152. 10.1152/ajpcell.2000.278.6.C1143 10837342

[acel13042-bib-0056] Tan, H. , Qurashi, A. , Poidevin, M. , Nelson, D. L. , Li, H. , & Jin, P. (2012). Retrotransposon activation contributes to fragile X premutation rCGG‐mediated neurodegeneration. Human Molecular Genetics, 21, 57–65. 10.1093/hmg/ddr437 21940752 PMC3235010

[acel13042-bib-0057] Tan, L. , & Shi, Y. G. (2012). Tet family proteins and 5‐hydroxymethylcytosine in development and disease. Development, 139, 1895–1902. 10.1242/dev.070771 22569552 PMC3347683

[acel13042-bib-0058] Tsumagari, K. , Baribault, C. , Terragni, J. , Varley, K. E. , Gertz, J. , Pradhan, S. , … Ehrlich, M. (2013). Early de novo DNA methylation and prolonged demethylation in the muscle lineage. Epigenetics, 8, 317–332. 10.4161/epi.23989 23417056 PMC3669123

[acel13042-bib-0059] Tsurumi, A. , & Li, W. X. (2012). Global heterochromatin loss: A unifying theory of aging? Epigenetics, 7, 680–688. 10.4161/epi.20540 22647267 PMC3414389

[acel13042-bib-0060] Villeponteau, B. (1997). The heterochromatin loss model of aging. Experimental Gerontology, 32, 383–394. 10.1016/S0531-5565(96)00155-6 9315443

[acel13042-bib-0061] Walsh, C. P. , Chaillet, J. R. , & Bestor, T. H. (1998). Transcription of IAP endogenous retroviruses is constrained by cytosine methylation. Nature Genetics, 20, 116–117. 10.1038/2413 9771701

[acel13042-bib-0062] Wilson, D. , Jackson, T. , Sapey, E. , & Lord, J. M. (2017). Frailty and sarcopenia: The potential role of an aged immune system. Ageing Research Reviews, 36, 1–10. 10.1016/j.arr.2017.01.006 28223244

[acel13042-bib-0063] Wu, W. , Ren, Z. , Wang, Y. , Chao, Z. , Xu, D. , & Xiong, Y. (2011). Molecular characterization, expression patterns and polymorphism analysis of porcine Six1 gene. Molecular Biology Reports, 38, 2619–2632. 10.1007/s11033-010-0403-9 21082258

[acel13042-bib-0064] Zykovich, A. , Hubbard, A. , Flynn, J. M. , Tarnopolsky, M. , Fraga, M. F. , Kerksick, C. , … Melov, S. (2014). Genome‐wide DNA methylation changes with age in disease‐free human skeletal muscle. Aging Cell, 13, 360–366. 10.1111/acel.12180 24304487 PMC3954952

