# Supplementary material for: Demethylation and derepression of genomic retroelements in the skeletal muscles of aged mice
Source: Aging Cell. 2019 Sep 27;18(6):e13042. doi: 10.1111/acel.13042 (PMC6826136; doi:10.1111/acel.13042)
Supplement: Supplementary file 1 [file ACEL-18-e13042-s001.docx]

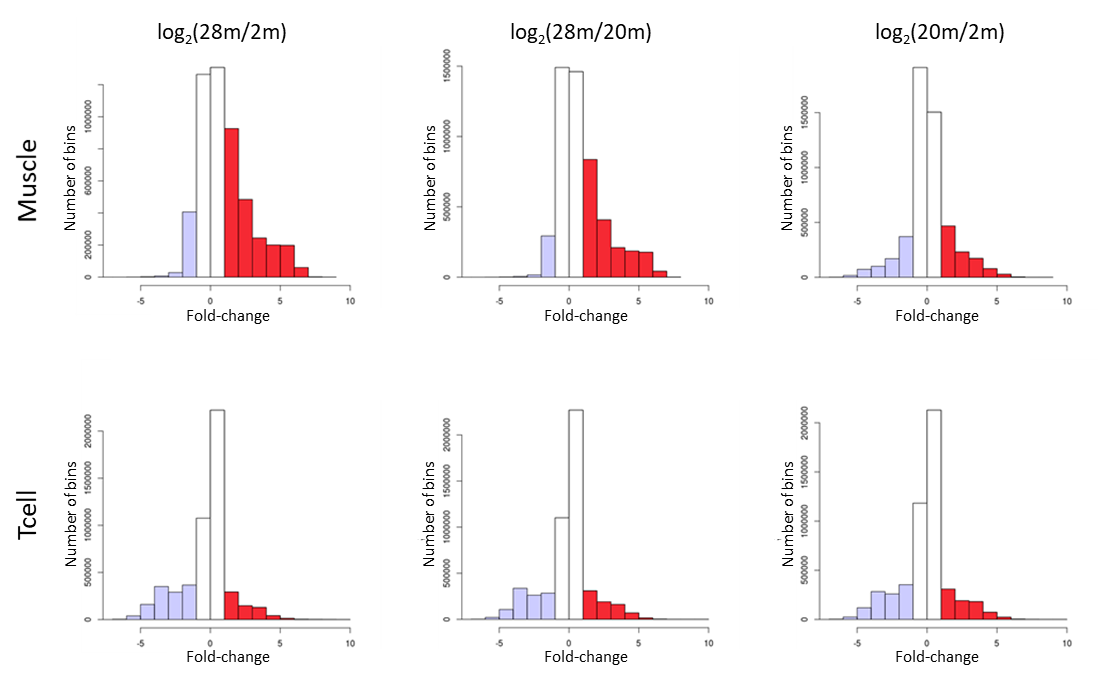


**Supplementary Figure S1** Histograms showing the distributions of genomic bins (500 bp) by log2-fold-change values between age groups.


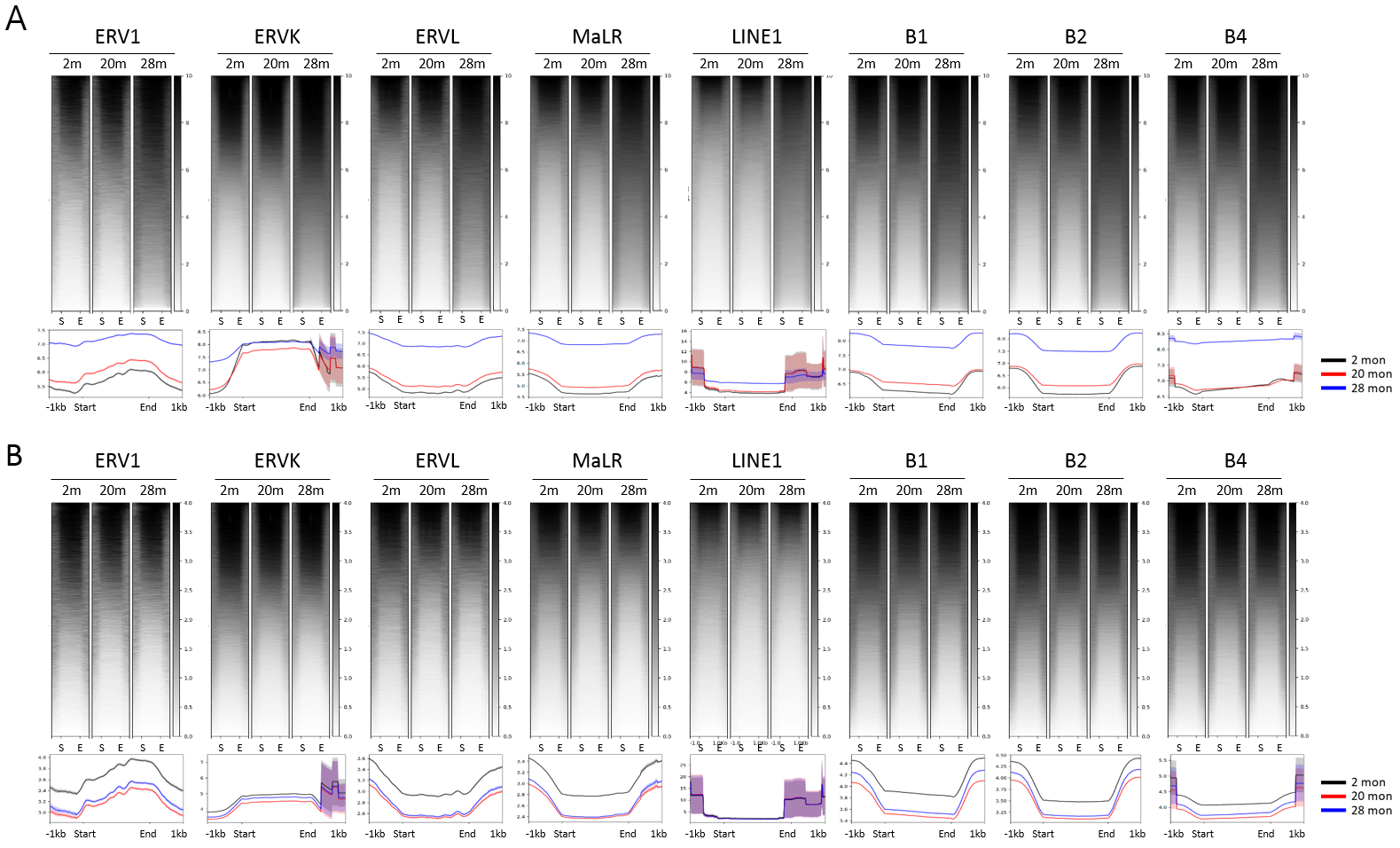


**Supplementary Figure S2** Heatmaps of DNA methylation densities within 1 kb of the start (S) and end (E) sites of entire copies in various types of retroelements in skeletal muscle (A) and T-cells (B). The stronger the color, the higher the DNA methylation levels. Mean DNA methylation levels in each age group were depicted in the profile plots (lower panels).


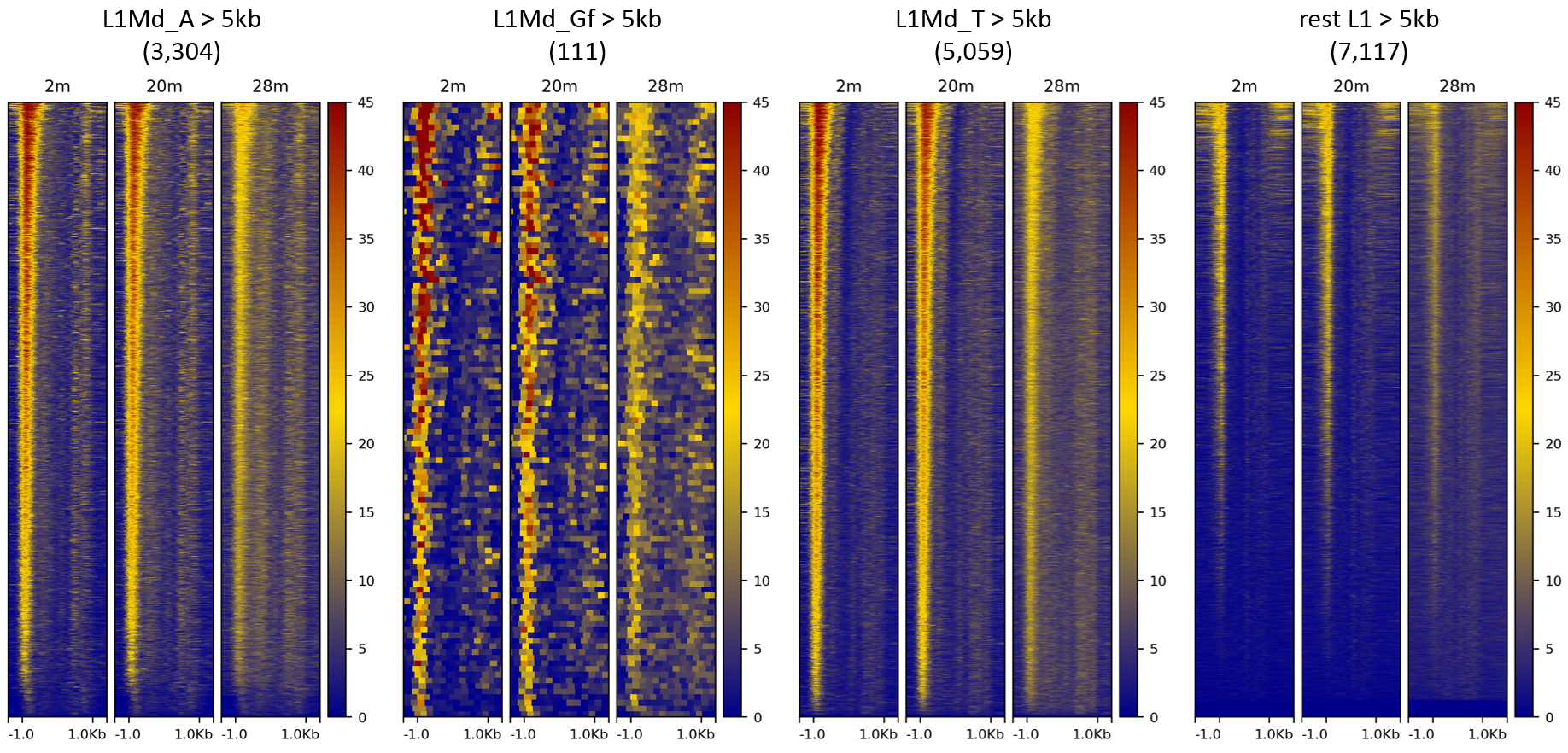


**Supplementary Figure S3** Heatmaps of DNA methylation densities of the evolutionarily active LINE1 families (L1Md_A, L1Md_Gf, and L1Md_T) and the rest of the LINE1 families (rest L1) in mouse skeletal muscle of 2, 20, and 28 months of age. The number of the members of each family is indicated in the parenthesis.

**
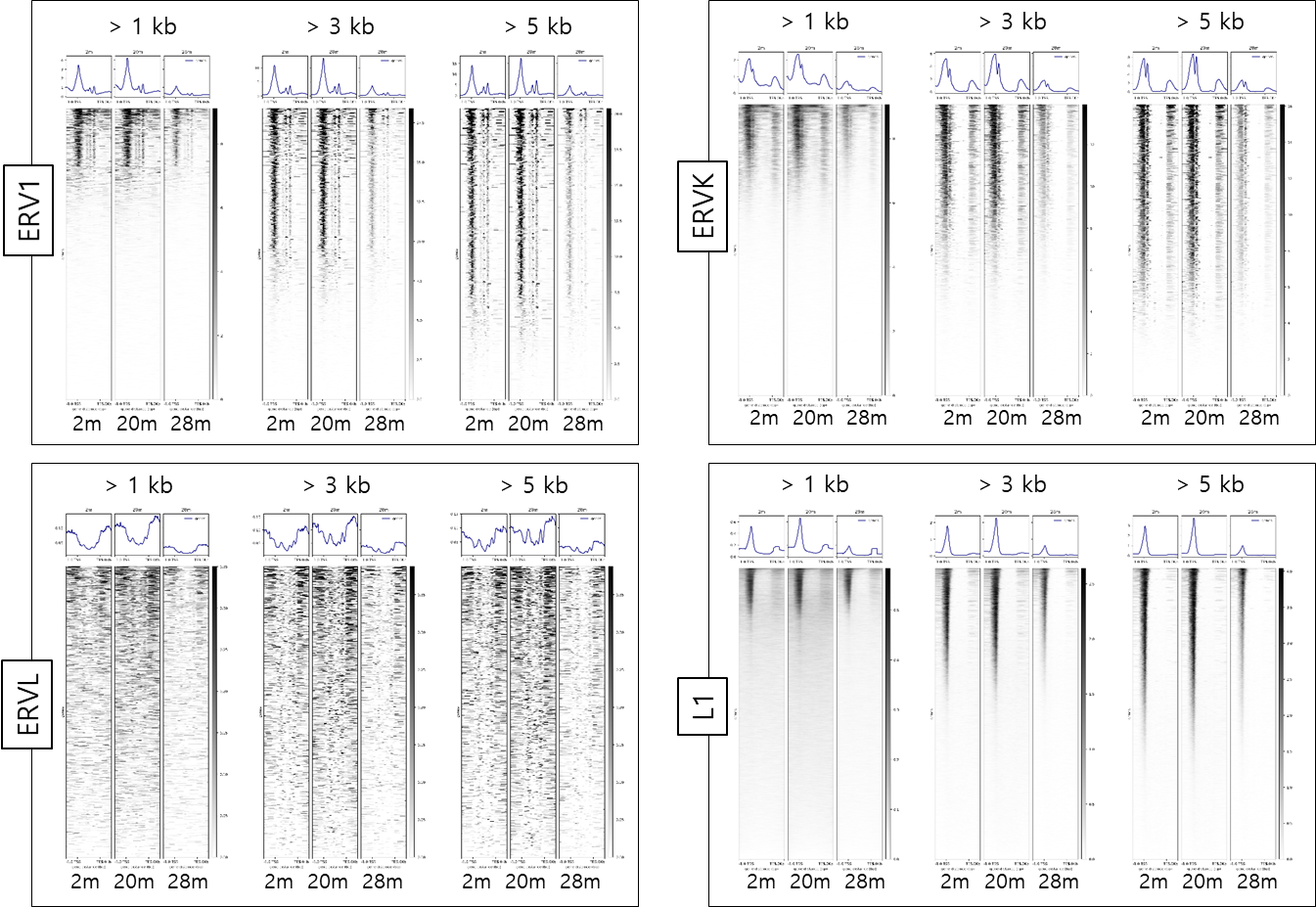
**

**Supplementary Figure S4** Heatmaps of DNA methylation densities of large ERV1, ERVK, ERVL, and LINE1 retroelement copies in skeletal muscle. Line graphs at the top of each heatmap illustrate the mean methylation levels of the retroelement copies at different ages.


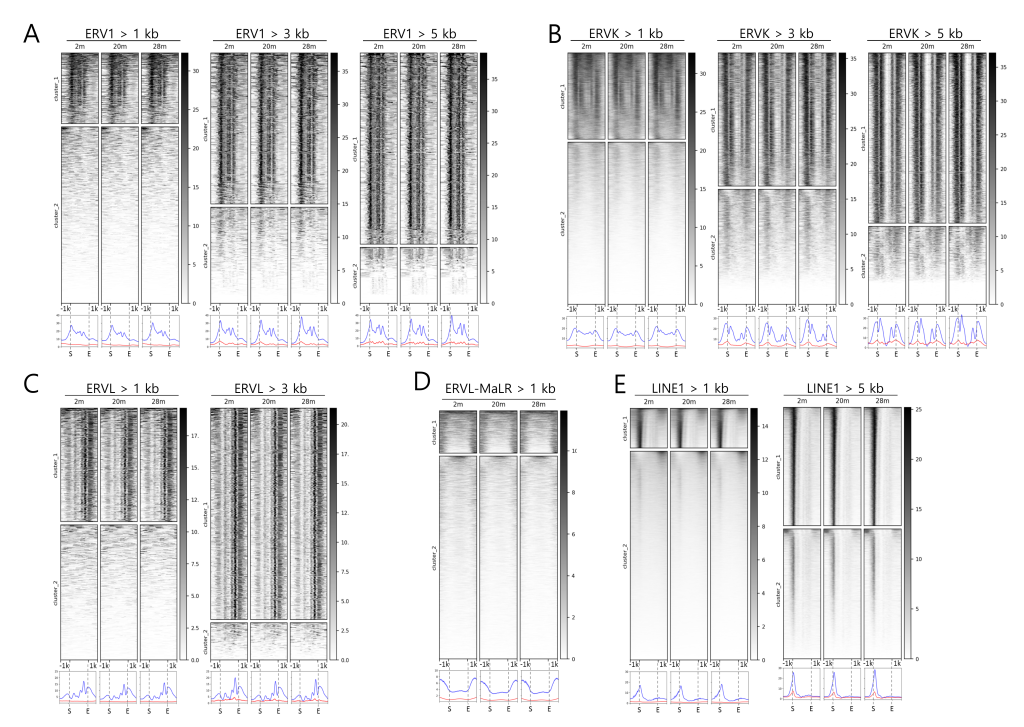


**Supplementary Figure S5** Heatmaps of DNA methylation densities of ERV1 (A), ERVK (B), ERVL (C), ERVL-MaLR (D), and LINE1 (E) in T cells. All groups were clustered by *k*-means algorithm (*k* = 2). Genomic copies of each retroelement family were grouped by length of > 1 kb, >3 kb, or >5 kb. Graphs in the bottom show the mean methylation levels of the two clusters (blue line for cluster-1 and red line for cluster-2) separately.


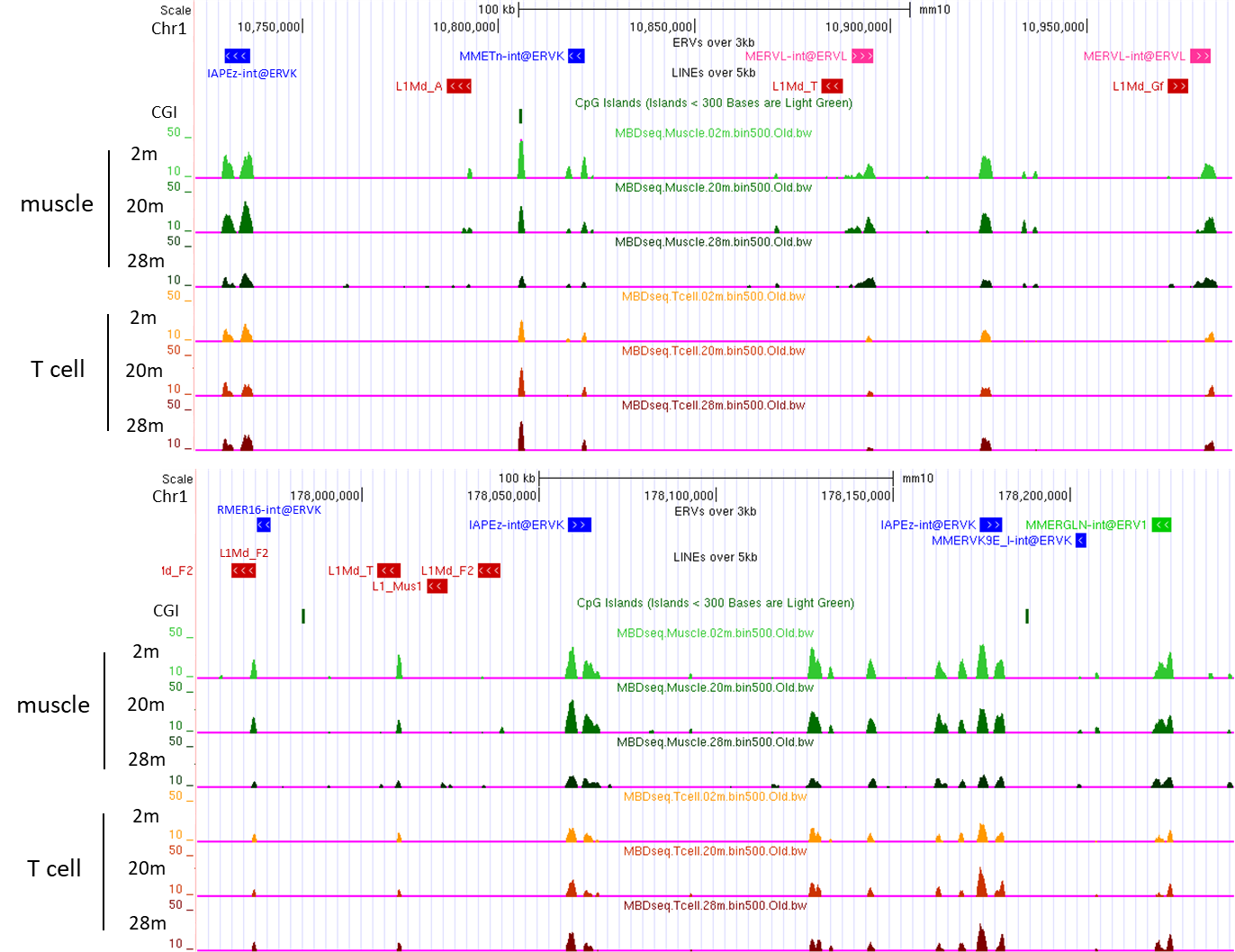


**Supplementary figure S6** Distinctive DNA methylation patterns in different retroelement families reproducibly appear in both skeletal muscle and T cells. The genome browser shows only the large-size LINE1 (red boxes; > 5.0 kb) and ERV (> 3.0 kb; green for ERV1, blue for ERVK, and pink for ERVL) copies extracted from the repeatMasker (mm10) track. Arrows in the retroelement boxes indicate the direction of the transcription.


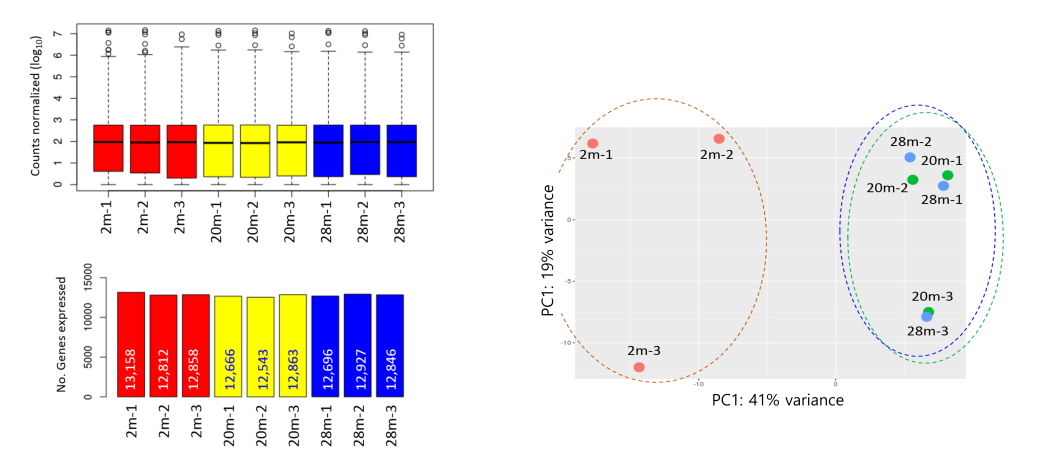


**Supplementary figure S7** Transcriptomic analysis of 2m, 20m, and 28m skeletal muscle (*n* = 3 each group). Count distributions among the muscle samples and the numbers of genes expressed in the samples (counts ≥10) are shown on the left. Principal component analysis (PCA) is presented on the right.

A

D

B


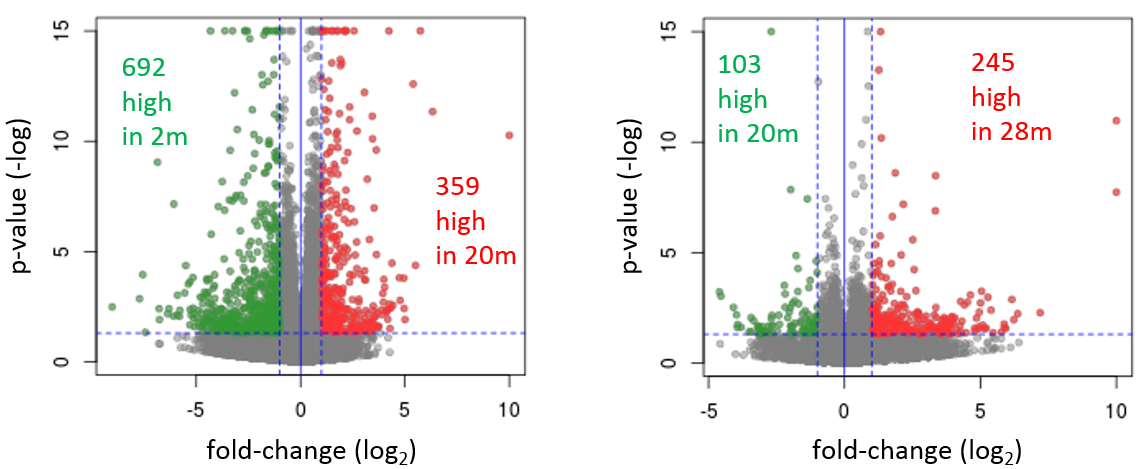


2m vs. 20m

20m vs. 28m


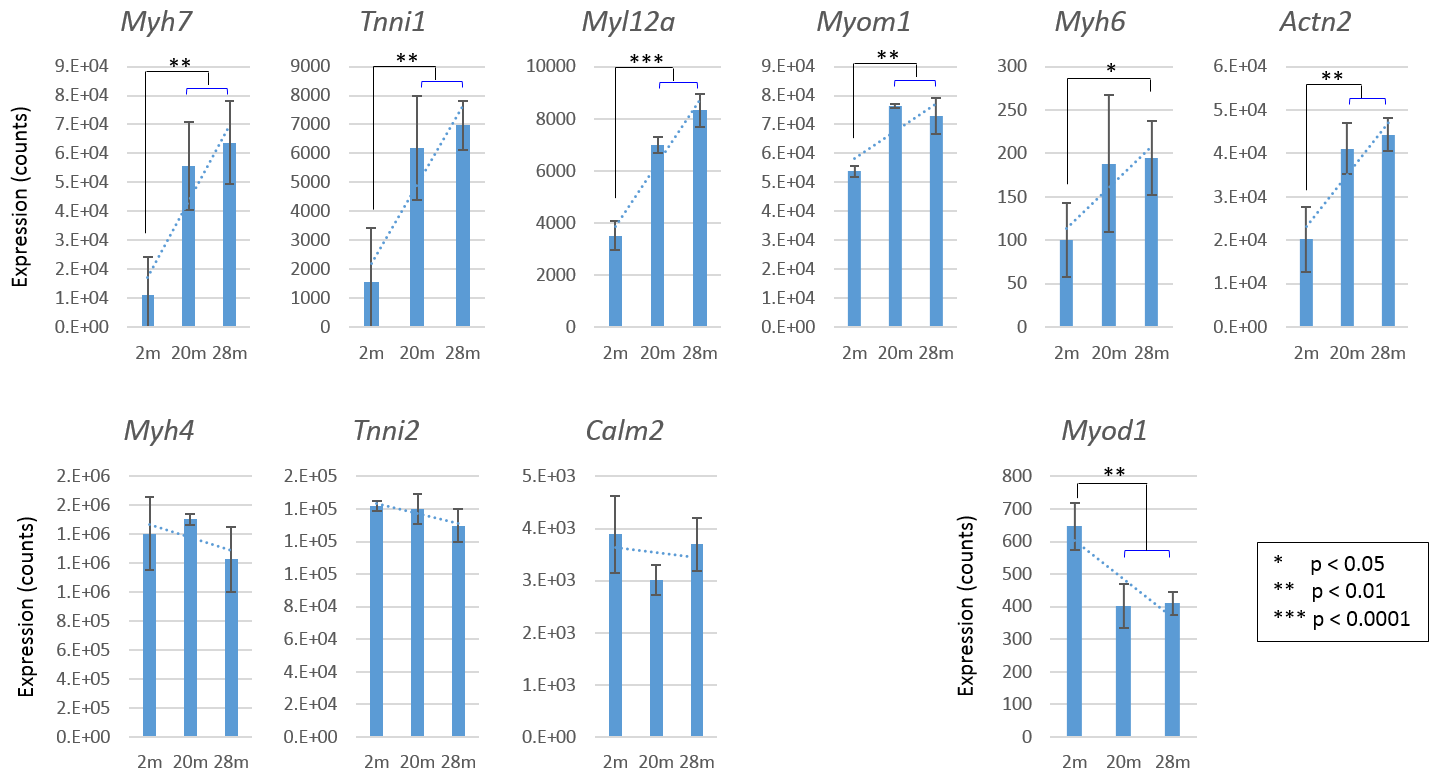


C

**Supplementary Figure S8** Expression levels of slow- (A), fast-twitch fiber genes (B), and of the *Myod1* gene (C) were compared between young (2m) and old (20m and 28m) skeletal muscle samples. Dotted lines in A–C indicate trend lines. Asterisks denote significant differences. Volcano plots in D present transcriptomic differences between the indicated age groups with highlighted differentially expressed genes (DEGs; fold change >2.0 and *p* < 0.05).


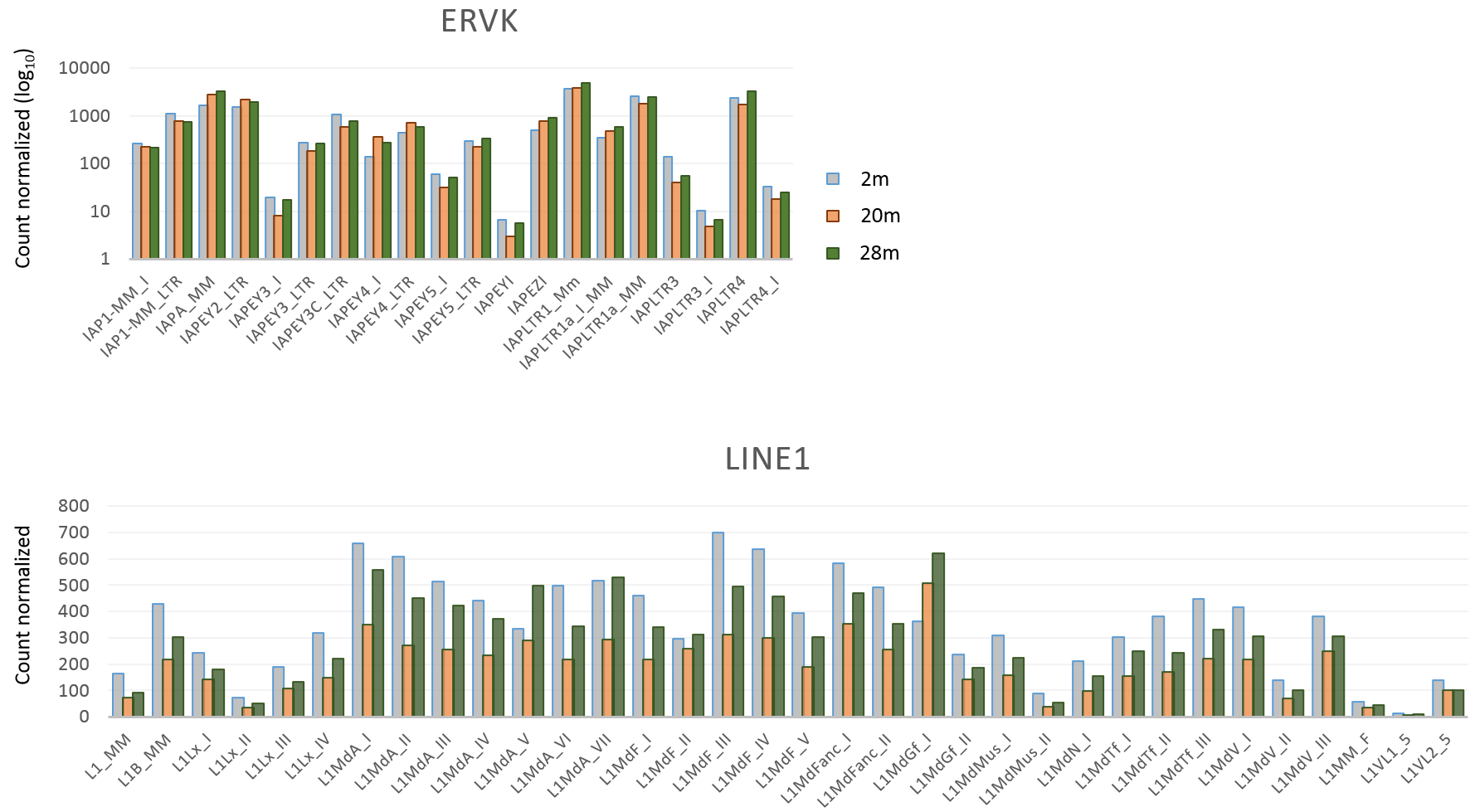


**Supplementary Figure S9** Age-associated changes in the transcript levels of various ERVK and LINE1 subfamilies in mouse skeletal muscle of 2, 20, and 28 months of age. Expression levels were measured using ‘SalmonTE’ program.


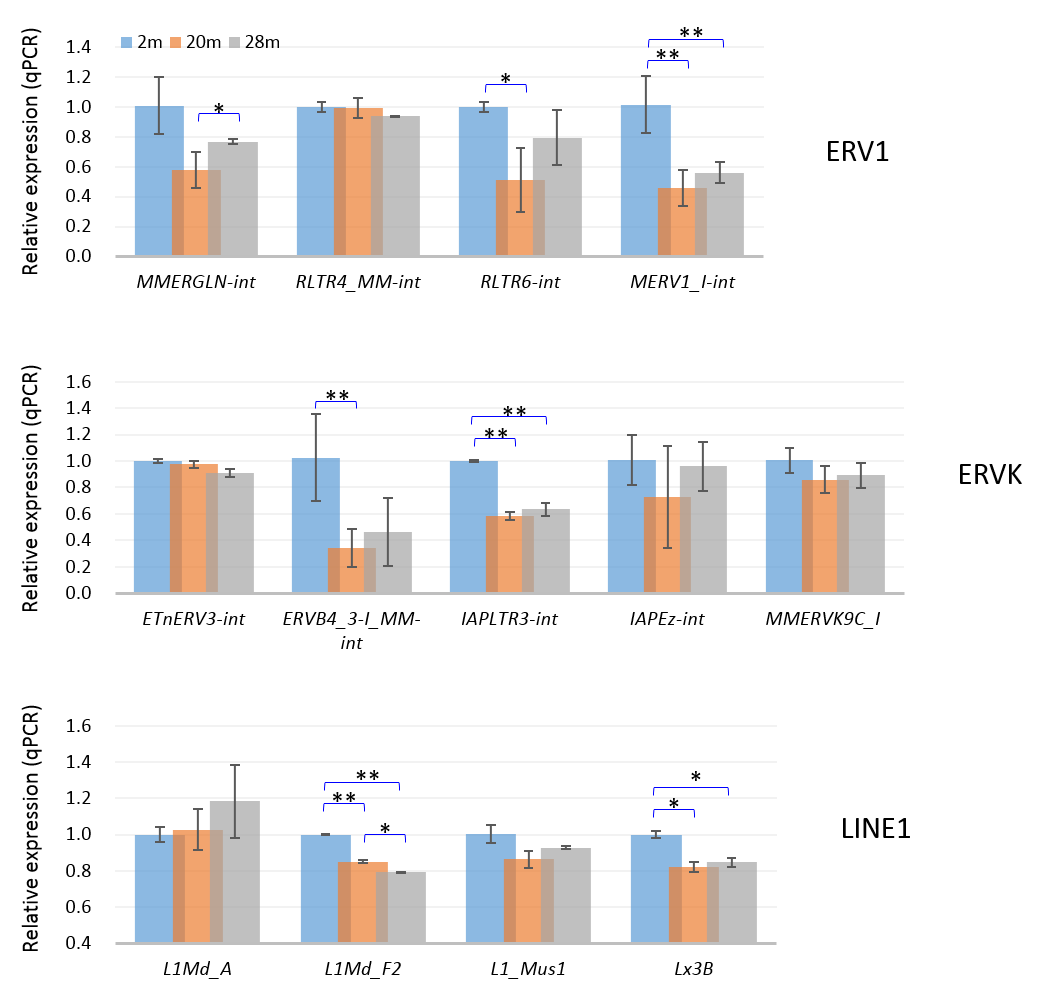

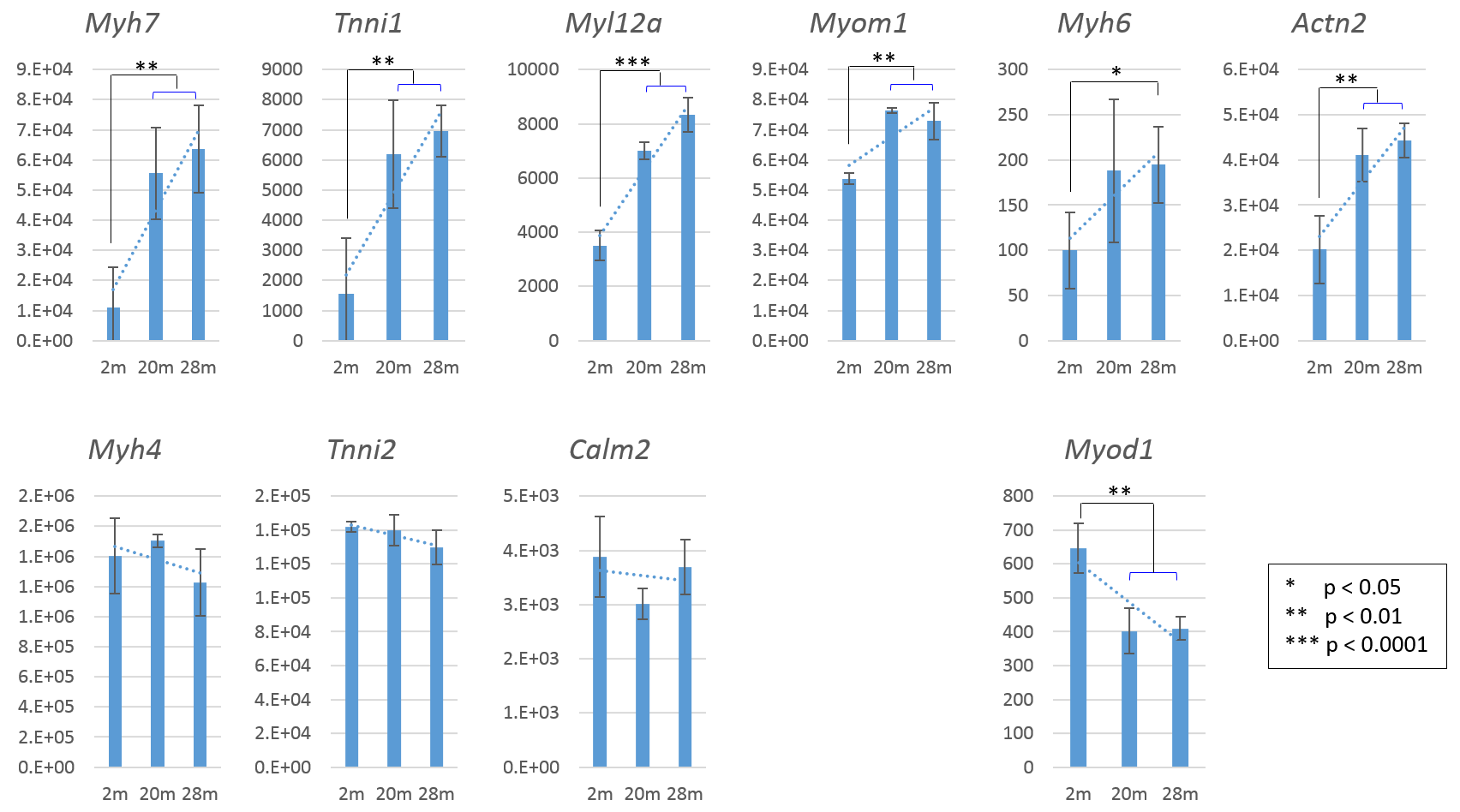


**Supplementary Figure S10** Quantitative real-time PCR analysis of expression of retroelement subfamilies in 2m, 20m, and 28m skeletal muscle. Asterisks indicate significant differences. The L1Md_F2, L1Md_Mus1, and Lx3B families were chosen by their relatively high read counts in the RNA-seq result (see Figure 5E).

Supplementary Figure S8
